# Traumatic Spinal Cord Injury and Subsequent Risk of Developing Chronic Cardiovascular, Neurologic, Psychiatric, and Endocrine Disorders

**DOI:** 10.1001/jamanetworkopen.2025.41157

**Published:** 2025-11-04

**Authors:** Ahmad Mashlah, Sandro Marini, Hunter Mills, Taha Yahya, Farid Radmanesh, Joshua Chalif, Benjamin E. Zusman, Joshua D. Bernstock, Maryam H. Al Mansi, Rachel Grashow, Muhammad M. Abd-El-Barr, Hasan A. Zaidi, Yi Lu, Ali Salim, Akl C. Fahed, Geoffrey T. Manley, Cathra Halabi, Anthony Digiorgio, Ross Zafonte, Saef Izzy

**Affiliations:** 1 Divisions of Stroke, Cerebrovascular, and Critical Care Neurology, Department of Neurology, Brigham and Women’s Hospital, Boston, Massachusetts; 2 Harvard Medical School, Boston, Massachusetts; 3 Bakar Computational Health Sciences Institute, University of California, San Francisco; 4 Weill Institute for Neurosciences, University of California, San Francisco; 5 Department of Neurosurgery, Division of Neurocritical Care, University of Texas Health Science Center, Houston; 6 Department of Neurosurgery, Brigham and Women’s Hospital, Boston, Massachusetts; 7 Department of Environmental Health, Harvard T.H. Chan School of Public Health, Boston, Massachusetts; 8 The Football Players Health Study at Harvard University, Boston, Massachusetts, Massachusetts; 9 Department of Neurosurgery, Duke University, Durham, North Carolina; 10 Trauma, Burn and Surgical Critical Care, Brigham and Women’s Hospital, Boston, Massachusetts; 11 Division of Cardiology, Department of Medicine, Massachusetts General Hospital, Boston; 12 Department of Neurological Surgery, University of California, San Francisco; 13 Institute for Health Policy Studies, University of California, San Francisco; 14 Department of Neurology, University of California, San Francisco; 15 Department of Physical Medicine and Rehabilitation, Spaulding Rehabilitation Hospital, Harvard Medical School, Boston, Massachusetts; 16 Department of Physical Medicine and Rehabilitation, University of Missouri, Columbia

## Abstract

**Importance:**

While acute complications of traumatic spinal cord injury (TSCI) are well characterized, long-term systemic sequelae remain poorly understood, particularly in previously healthy individuals.

**Objective:**

To evaluate the long-term risk of neurologic, psychiatric, cardiovascular, and endocrine comorbidities and associated mortality in patients with TSCI compared with matched uninjured controls.

**Design, Setting, and Participants:**

This retrospective cohort study analyzed longitudinal data from 2 large hospital-based registries (January 1996 to January 2024): Mass General Brigham (MGB) and the University of California (UC) Health System. Individuals with preexisting diagnoses of studied comorbidities were excluded. Uninjured controls were matched 3:1 by age, sex, and race. The data were analyzed in September to December 2024.

**Exposure:**

TSCI.

**Main Outcomes and Measures:**

Incidence of cardiovascular, endocrine, neurologic, and psychiatric conditions, as defined by *International Classification of Diseases, Ninth Revision* (*ICD-9*) or *Tenth Revision* (ICD-10). Associations between TSCI and comorbidities, and their association with all-cause mortality, were evaluated using multivariable Cox and logistic regression models.

**Results:**

A total of 1038 patients with TSCI in the MGB cohort (602 [58%] male; median [IQR] age, 44 [31-59] years) and 1711 in the UC cohort (1111 [65%] male; median [IQR] age, 45 [32-58] years) were matched with 3114 uninjured control participants in MGB (median [IQR] age, 43 [31-59] years) and 5133 uninjured control participants in UC (median [IQR] age, 45 [31-60] years). Compared with controls, patients with TSCI had significantly higher risk of hypertension (hazard ratio [HR], 1.6; 95% CI, 1.3-1.9), hyperlipidemia (HR, 1.5; 95% CI, 1.3-1.8), ischemic stroke (HR, 2.5; 95% CI, 1.7-3.7), coronary artery disease (HR, 1.8; 95%CI: 1.3-2.5), and diabetes (HR, 1.5; 95% CI, 1.1-2.1), along with other neurologic and psychiatric conditions. The risk of post-TSCI comorbidities was elevated across different age groups, including patients aged 18 to 45 years compared with age-matched controls, with higher rates of hypertension (HR 1.5, 95% CI 1.1-2.1) and ischemic stroke (HR 2.8, 95% CI 1.3-6.0) observed in the TSCI group. Similar findings were seen in a separate cohort of 1711 patients with TSCI from UC Health. Postinjury hypertension (odds ratio [OR], 2.0; 95% CI, 1.2-3.5), pituitary dysfunction (OR, 6.5; 95% CI, 1.1-33.2), adrenal insufficiency (OR, 5.0; 95% CI, 1.04-20.2), depression (OR, 2.9; 95% CI, 1.6-5.2), substance misuse (OR, 4.0; 95% CI, 1.5-9.8), seizures (OR, 6.4; 95% CI, 2.7-14.5), and dementia (OR, 4.8; 95% CI, 2.0-11.6) were associated with higher mortality.

**Conclusions and Relevance:**

In this cohort study, TSCI was associated with increased long-term risk of multisystem morbidity and elevated mortality—even in previously healthy patients. These findings underscore the need for proactive, longitudinal, and multidisciplinary approaches to prevent complications and protect this high-risk population.

## Key Points

**Question:**

Is traumatic spinal cord injury (TSCI) associated with increased long-term risk of developing neurologic, psychiatric, cardiovascular, and endocrine comorbidities in previously healthy individuals?

**Findings:**

In this cohort study of 2749 patients with TSCI and 8247 uninjured control participants, TSCI was associated with a significantly increased risk of developing major chronic conditions across neurologic, psychiatric, cardiovascular, and endocrine domains, with hazard ratios ranging from 1.5 to 2.5, with elevated risks evident across all age groups and spinal injury sites, including among individuals younger than 45 years. Post-TSCI comorbidities were also associated with increased mortality.

**Meaning:**

These results underscore the need for long-term, multidisciplinary care strategies—including proactive screening and management programs—for individuals with TSCI, even in the absence of preexisting conditions, to address the heightened risk of systemic comorbidities and associated mortality.

## Teaser

This cohort study examines whether individuals with traumatic spinal cord injury have greater risk of neurologic, psychiatric, cardiovascular, and endocrine comorbidities compared with their uninjured counterparts.

## Introduction

Traumatic spinal cord injuries (TSCI) are a major global health concern and cause significant long-term disability and mortality, leading to substantial burdens on health care systems.^1,2^ Even after accounting for mortality in the acute phase, survival rates after TSCI are significantly lower than in age-matched uninjured individuals.^3,4^ Recent studies showed that traumatic brain injury (TBI) has significant long-term clinical consequences, with increased risk of cardiovascular, endocrine, neurological, and psychiatric diseases.^5,6,7,8,9,10,11^ It is therefore possible that TSCI can similarly increase the risk of systemic diseases, which in turn can contribute to increased morbidity and mortality observed in this vulnerable population. Few observational studies have investigated this hypothesis^12^: Huang et al^13^ found an increased risk of dementia after TSCI in a prospective study of a Taiwanese cohort. Other studies showed that TSCI was associated with increased odds of cardiovascular disease, including myocardial infarction, heart failure, atrial fibrillation,^12^ and stroke.^14^ However, prior studies have been limited by short follow-up durations, inefficient patient-level data, inclusion of individuals with preexisting comorbidities, or restriction to veteran populations. A more comprehensive understanding of post-TSCI sequelae is essential to inform targeted prevention and intervention strategies aimed at mitigating the substantial long-term health burden associated with this condition. We present findings from 2 large, multicenter longitudinal cohorts in the United States, offering a comprehensive assessment of the long-term incidence of chronic neurologic, psychiatric, cardiovascular, and endocrine disorders in previously healthy individuals with TSCI, compared with age-, sex-, and race-matched uninjured controls.

## Methods

This cohort study used 2 patient repositories: (1) the Mass General Brigham (MGB) Research Patient Data Registry (RPDR), a web-based tool that enables querying a centralized clinical data repository with information on inpatient, outpatient, and emergency department encounters; and (2) UC Health Data Warehouse, which uses electronic health records from 6 sites from the University of California (UC): Davis, Irvine, Los Angeles, Riverside, San Diego, and San Francisco.^5^ Data were analyzed separately at each institute to maintain security provisions attached to data use. This approach prevented us from combining the 2 datasets. Analyzing both datasets provided a broader characterization of patients after TSCI across different health care settings and geographic regions. For both cohorts, the institutional review boards waived formal review and informed consent because the study did not meet the definition of human participant research. The study was conducted following the Strengthening the Reporting of Observational Studies in Epidemiology (STROBE) reporting guideline for cohort studies.

### Patients, Control Participants, and Comorbidity Selection

We applied the same methods to both MGB and UC cohorts. In brief, we queried all patients with TSCI from January 1996 to January 2024. We excluded patients younger than 18 years and those with no encounters before the TSCI index date. We also excluded those who had any of the 21 studied comorbidities (eTable 1 in Supplement 1) prior to or within 1 year after the index date to exclude undiagnosed comorbidities at time of injury. For both MGB and UC, we identified the uninjured control group by frequency-matching individuals in the same repository at a 3:1 ratio. Control participants were matched for age, sex, and race, and the same exclusion criteria were applied. Race was included in the matching process to minimize the influence of demographic disparities on observed outcomes.^15,16^ The index date was defined as the first TSCI diagnosis date for the injured groups. For the control participants, the index date was a random hospital or outpatient clinic encounter date, to minimize temporal clustering. Age was defined by age on the index date. Race and ethnicity data were collected from medical records or self-reported by patients using standardized options, as previously reported.^6^ Briefly, the options consisted of American Indian or Alaska Native, Asian, Black, Hispanic, White, and other, or those with missing racial information. The option other was defined as Asian Pacific Islander, Hawaiian, and Middle Eastern, and those who selected multiple races or ethnicities.

### Exposure

TSCI was defined using the US Centers of Disease Control and Prevention criteria and extracted using the validated *International Classification of Diseases, Ninth Revision* (*ICD-9*) and *Tenth Revision* (*ICD-10*)^17^ (eTable 1 in Supplement 1). The external cause of injury was categorized as due to fall, motor vehicle collision, firearm, or unspecified.

### Outcome

We studied 21 diseases across 4 organ systems. These included: (1) cardiovascular diseases: hypertension, hyperlipidemia, obesity, and coronary artery disease; (2) endocrine disorders: hypothyroidism, pituitary dysfunction, diabetes, adrenal insufficiency, and erectile dysfunction; (3) neurological disorders: ischemic stroke or transient ischemic attack, seizure, and dementia; and (4) psychiatric and substance use disorders: depression; bipolar disorder; psychosis; anxiety disorder; sleep disorder; suicide ideation, intent, or attempt; substance misuse; opioid misuse; and alcohol misuse. The diagnoses were determined using *ICD-9* or *ICD-10* codes^6^ (eTable 1 in Supplement 1). Disease onset date was defined as the date when a diagnosis was first documented in the medical record. We additionally analyzed mortality risk following disease diagnosis in the MGB cohort (mortality data not available in UC cohort).

### Statistical Analysis

We describe both cohorts using proportions and medians with IQRs, as appropriate. Cox proportional hazards models, adjusted for age, sex, and race, were used to compute hazard ratios (HRs) and 95% CIs of the associations of TSCI with chronic diseases. Patients were censored at time of disease diagnosis, death, or last available encounter, whichever occurred first. The risk window extended from 1-year after the index encounter until the last encounter recorded. Schoenfeld residuals were used to evaluate the proportional hazards assumption, which was met in most instances except for hyperlipidemia, sleep disorder and suicidal ideation, intent, or attempt in the MGB cohort (*P* = .04; *P* = .04; and *P* = .02, respectively) and coronary artery disease in the UC cohort (*P* = .04).

Kaplan-Meier plots demonstrated the cumulative risk of each disease in TSCI and uninjured groups with between-group comparisons by log-rank test. Additionally, we used interaction terms to explore the interaction of age and TSCI on subsequent disease risk. We performed sensitivity analyses including traumatic brain injury (TBI) as a model covariate or stratified by spinal injury location (cervical, thoraco-lumbar), and age (stratified to 3 age categories of 18-45, 46-65, and >65 years).

In MGB, logistic regression assessed the association of each disease with mortality in the TSCI group. To capture the true association of post-TSCI comorbidities with long term mortality, the risk of mortality window extended from 3 years after the injury until the last recorded encounter. This time span was chosen based on the preliminary analysis showing the median time from the injury to disease onset.

To examine potential bias that might have been introduced by differences in the number of encounters before the comorbidity diagnosis, we compared the median number of encounters before each diagnosis between the uninjured and TSCI groups using the Wilcoxon rank-sum test.^6^ Additional sensitivity analyses were performed by restricting the cohort to patients indexed before 2015 to ensure sufficient follow-up and by censoring follow-up at 15 years to mitigate potential loss-to-follow-up bias. We also conducted a sensitivity analysis using restricted mean survival time instead of hazard ratios to validate the robustness of our findings.

We defined α as .05 and adjusted for multiple comparisons using Bonferroni correction (*P* < .002)^18^ for the primary analysis and *P* < .05 for the secondary analyses. All analyses were performed using RStudio version 4.3.1 (R Project for Statistical Computing). The data were analyzed in September to December 2024.

## Results

### Demographics

We identified a total of 18 644 patients with TSCI in the MGB cohort and 12 311 in the UC cohort between 1996 and 2024. Of these, 1038 patients with TSCI (602 [58%] male; 66 [6%] Black, 37 [4%] Hispanic, and 796 [77%] White; median [IQR] age, 44 [31-59] years) from MGB and 1711 patients (1111 [65%] male; 133 [8%] Black, 307 [18%] Hispanic, and 936 [55%] White; median [IQR] age, 45 [32-58] years) from UC cohort met the inclusion and exclusion criteria for the study (eFigure 1 in Supplement 1). Using the larger MGB RPDR and UC system data pools, we selected an age-, sex-, and race-frequency–matched uninjured group without a diagnosis of TSCI or other outcome diagnosis (MGB RPDR, median [IQR] age, 43 [31-59] years; UC system, median [IQR] age, 45 [31-60] years) who were registered between 1996 and 2024.

In MGB, the mechanism of injury was not available for all participants, but motor vehicle collision was the most common mechanism (178 [17%]) in those with available data. The mechanisms of TSCI were not available for the UC cohort. For both cohorts, the most common level of injury was cervical, observed in 381 patients (37%) and 673 patients (39%) in MGB and UC, respectively. In both cohorts, the median number of encounters among patients with TSCI before the index date was similar to the uninjured group (Table). However, patients with TSCI had a higher number of follow-up encounters compared with control participants after the index date in both cohorts (MGB: median [IQR], 17 [7-51] visits vs 10 [5-26] visits; and UC: 37 [15-86] visits vs 14 [5-35] visits).

**Table.** Baseline Patient Characteristics of MGB and UC Cohorts

Characteristic	Participants, No. (%)					
	MGB cohort			UC cohort		
	Overall (n = 4152)	Control (n = 3114)	TSCI (n = 1038)	Overall (n = 6844)	Control (n = 5133)	TSCI (n = 1711)
Sex						
Female	1744 (42)	1308 (42)	436 (42)	2400 (35)	1800 (35)	600 (35)
Male	2408 (58)	1806 (58)	602 (58)	4444 (65)	3333 (65)	1111 (65)
Age at TSCI, median (IQR), y	43 (31-59)	43 (31-59)	44 (31-59)	45 (31-59)	45 (31-60)	45 (32-58)
Age group, y						
18-45	2256 (54)	1701 (55)	555 (53)	3307 (48)	2488 (48)	819 (48)
46-65	1239 (30)	914 (29)	325 (31)	2459 (36)	1799 (35)	660 (39)
>65	657 (16)	499 (16)	158 (15)	1078 (16)	846 (16)	232 (14)
Race						
American Indian or Alaska Native	10 (<1)	5 (<1)	5 (<1)	28 (<1)	21 (<1)	7 (<1)
Asian	150 (4)	114 (4)	36 (4)	536 (8)	402 (8)	134 (8)
Black	271 (7)	205 (7)	66 (6)	532 (8)	399 (8)	133 (8)
Hispanic	117 (3)	80 (3)	37 (4)	1288 (18)	921 (18)	307 (18)
White	3213 (77)	2417 (78)	796 (77)	3744 (55)	2808 (55)	936 (55)
Unknown	391 (9)	293 (9)	98 (9)	776 (11)	582 (11)	194 (11)
Level of injury						
Cervical	NA	NA	381 (37)	NA	NA	673 (39)
Thoraco-lumbar	NA	NA	290 (28)	NA	NA	654 (38)
Unclassified	NA	NA	367(35)	NA	NA	384 (22)
TSCI mechanism						
Motor vehicle collision	NA	NA	178 (17)	NA	NA	NA
Fall	NA	NA	157 (15)	NA	NA	NA
Firearm	NA	NA	6 (<1)	NA	NA	NA
Unspecified	NA	NA	697 (67)	NA	NA	NA
TBI before index or concurrent at the index	430 (10)	176 (6)	254 (24)	144 (2)	22 (<1)	122 (7)
Encounters after index, median (IQR), No.	12 (5-30)	10 (5-26)	17 (7-51)	17 (6-46)	14 (5-35)	37 (15-86)
Encounters before index, median (IQR), No.	3 (1-8)	3 (1-7)	4 (2-12)	4 (2-10)	4 (2-10)	5 (2-12)
Follow up duration, median (IQR), y	6 (2-13)	6 (2-12)	5 (1-13)	4 (2-7)	4 (2-7)	4 (2-7)

Abbreviations: MGB, Mass General Brigham; NA, not applicable; TBI, traumatic brain injury; TSCI, traumatic spinal cord injury; UC, University of California.

### Multisystem Morbidity After TSCI

**Figure 1.**
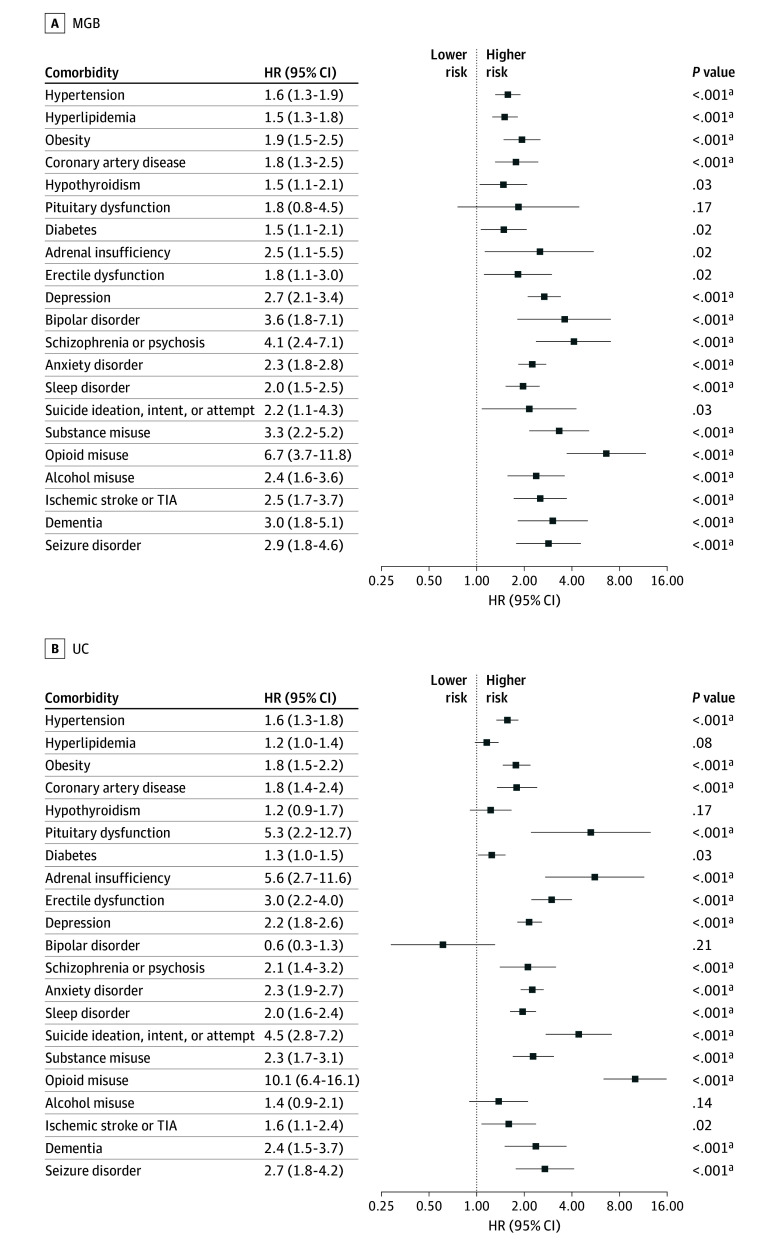
Risks of Multisystem Comorbidities After Traumatic Spinal Cord Injury in the Mass General Brigham (MGB) and University of California (UC) Cohorts HR indicates hazard ratio; TIA, transient ischemic attack. ^a^Statistically significant after Bonferroni correction.

**Figure 2.**
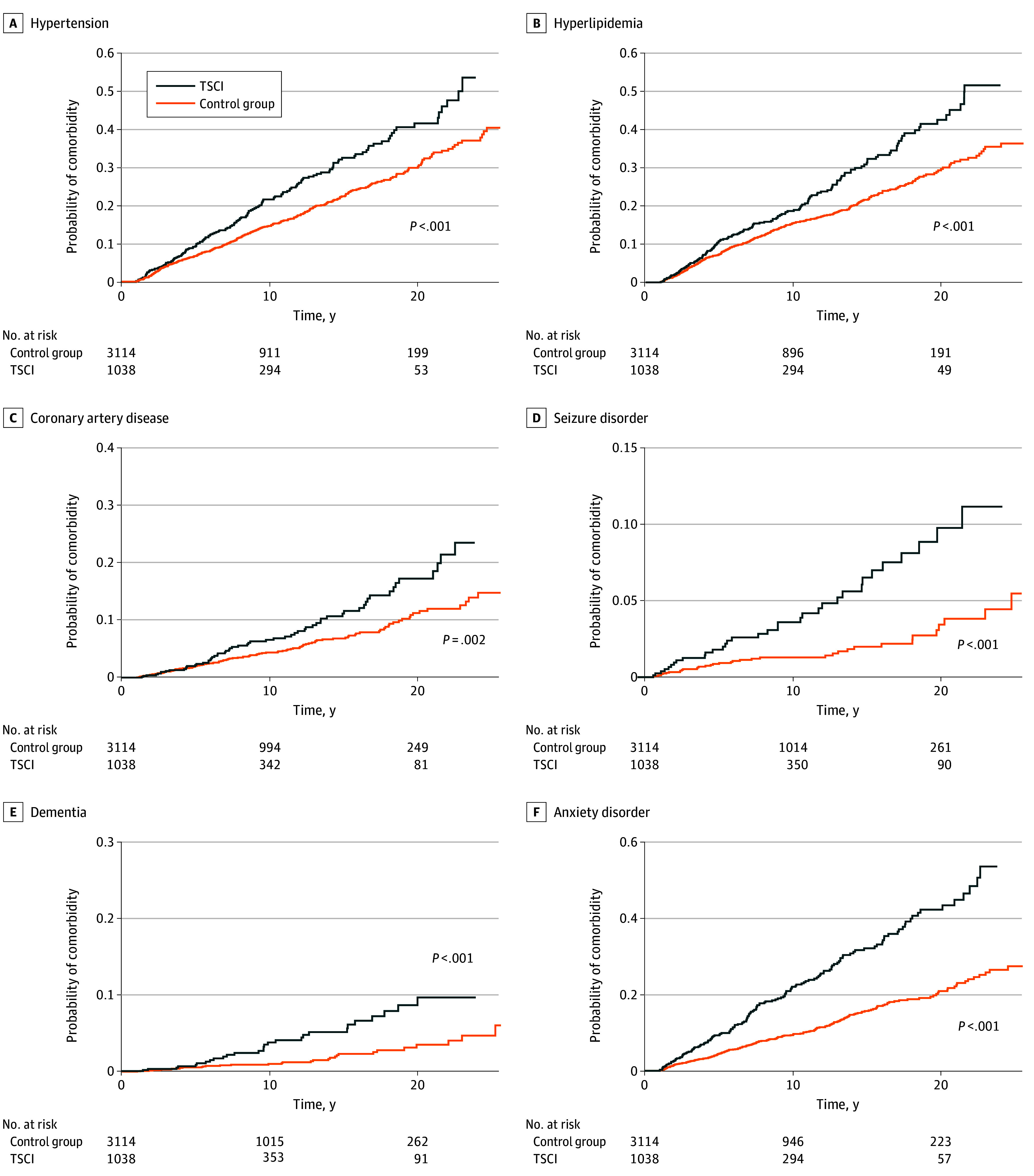
Kaplan-Meier Curves of Risk of Multisystem Comorbidities After Traumatic Spinal Cord Injury in the Mass General Brigham Cohort TSCI indicates traumatic spinal cord injury.

TSCI was associated with significantly increased risk of long-term multisystem comorbidities (Figures 1 and 2; eFigures 2-3 in Supplement 1). Across both cohorts, individuals with TSCI had a higher risk of cardiovascular diseases and associated risk factors, including hypertension (MGB: HR, 1.6; 95% CI, 1.3-1.9; UC: HR, 1.6; 95% CI, 1.3-1.8), and coronary artery disease (MGB: HR, 1.8; 95% CI, 1.3-2.5; UC: HR, 1.8; 95% CI, 1.4-2.4) compared with uninjured controls (Figure 1). An elevated risk of hyperlipidemia was observed in the MGB cohort (HR, 1.5; 95% CI, 1.3-1.8) but not in the UC cohort (HR 1.2; 95% CI, 1.0-1.4).

TSCI was also associated with increased risk of neurological disease in in both cohorts, including ischemic stroke (MGB: HR, 2.5; 95% CI, 1.7-3.7; UC: HR, 1.6; 95% CI, 1.1-2.4), dementia (MGB: HR, 3.0; 95% CI, 1.8-5.1; UC: HR, 2.4; 95% CI, 1.5-3.7), and seizures (MGB: HR, 2.9; 95% CI, 1.8-4.6; UC: HR, 2.7; 95% CI, 1.8-4.2). Psychiatric conditions were more prevalent in the TSCI group, including depression (MGB: HR, 2.7; 95% CI, 2.1-3.4; UC: HR, 2.2; 95% CI, 1.8-2.6). The risk of opioid use disorder was elevated in both cohorts.

Finally, TSCI was associated with a higher risk of endocrine disorders, although some estimates had wider confidence intervals. These included erectile dysfunction (MGB: HR, 1.8; 95% CI, 1.1-3.0; UC: HR, 3.0; 95% CI, 2.2-4.0) and adrenal insufficiency (MGB: HR, 2.5; 95% CI, 1.1-5.5; UC: HR, 5.6; 95% CI, 2.7-11.6) (eFigures 2-3 in Supplement 1).

Given the higher number of follow-up visits among patients with TSCI, we performed a sensitivity analysis to assess whether increased health care utilization was associated with greater detection of comorbidities. We compared the median number of medical encounters prior to the diagnosis of each comorbidity between the TSCI and control participants. For most of the outcomes evaluated, there was no significant difference in the median number of encounters between groups (eTable 2 in Supplement 1). Findings remained consistent in patients indexed prior to 2015 and in models with follow-up censored at 15 years (eTables 3-4 in Supplement 1), and in analyses using restricted mean survival time. Lastly, in both cohorts, adjustment for concomitant TBI did not modify the associations between TSCI and risk of long-term multisystem comorbidities (eTables 5-6 in Supplement 1).

### Age Stratification and Risk of Comorbidities

**Figure 3.**
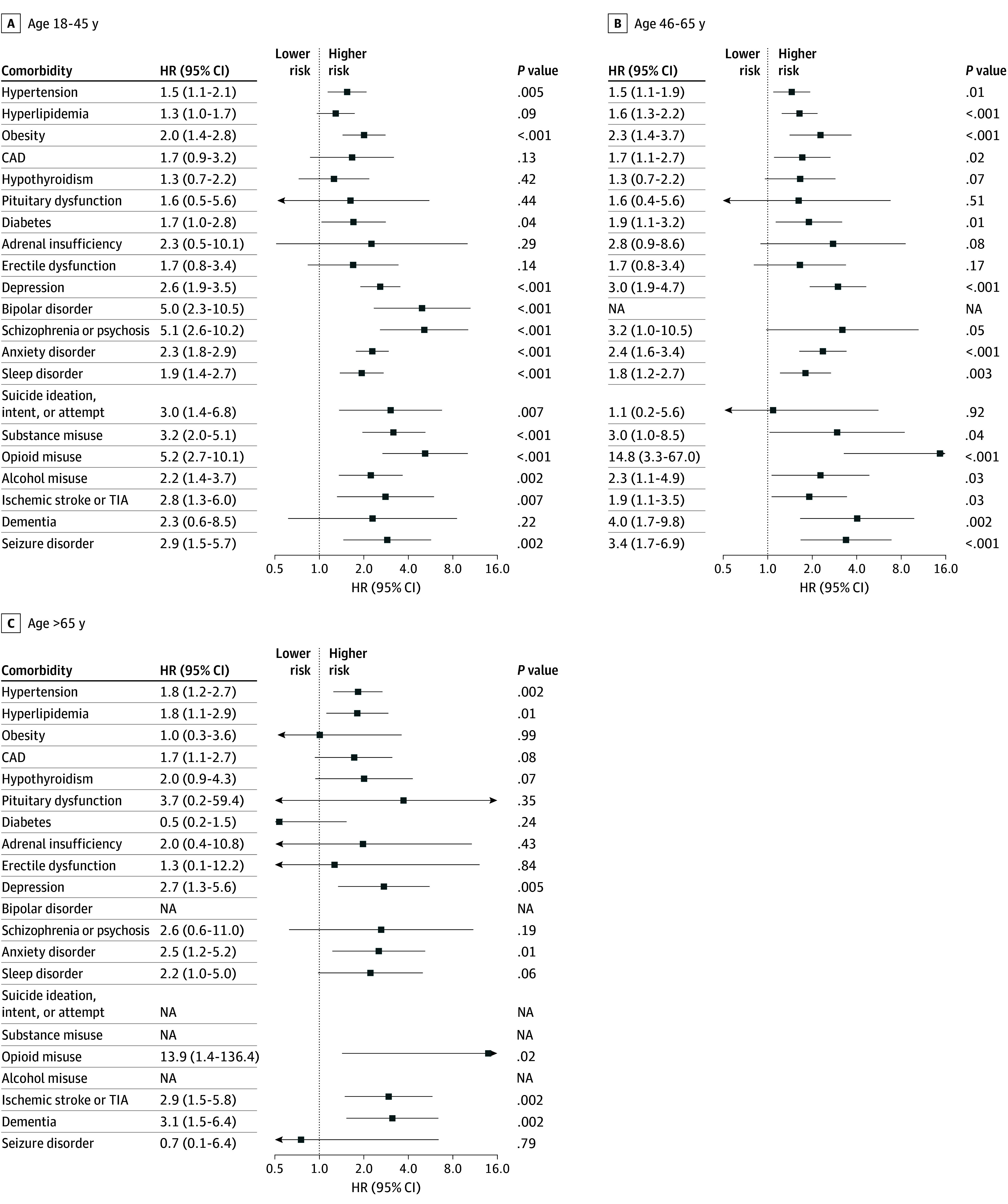
Risk of Multisystem Comorbidities After Traumatic Spinal Cord Injury, Stratified by Age in the Mass General Brigham Cohort CAD indicates coronary artery disease; HR, hazard ratio; NA, not applicable; TIA, transient ischemic attack.

For most disorders, the association of TSCI with chronic comorbidities was similar across age groups (Figure 3; eTables 7-8 in Supplement 1). This was consistent with an exploratory model including a TSCI by age interaction term which found no significant interaction between age and injury with regard to developing any chronic comorbidities in the MGB cohort (eTable 9 in Supplement 1). Exceptions included seizure disorder, which in both cohorts was only associated with TSCI in patients younger than 66 years, and bipolar disorder, which in the MGB cohort was increased in patients with TSCI aged 18 to 45 years and unanalyzable in other age groups due to the low number of such diagnosis in these age groups; in the UC cohort, there was no association between bipolar disorder and TSCI across age groups, and the comparison for patients older than 65 years was not analyzable due to the absence of cases.

### Spinal Injury Location and Risk of Comorbidities

**Figure 4.**
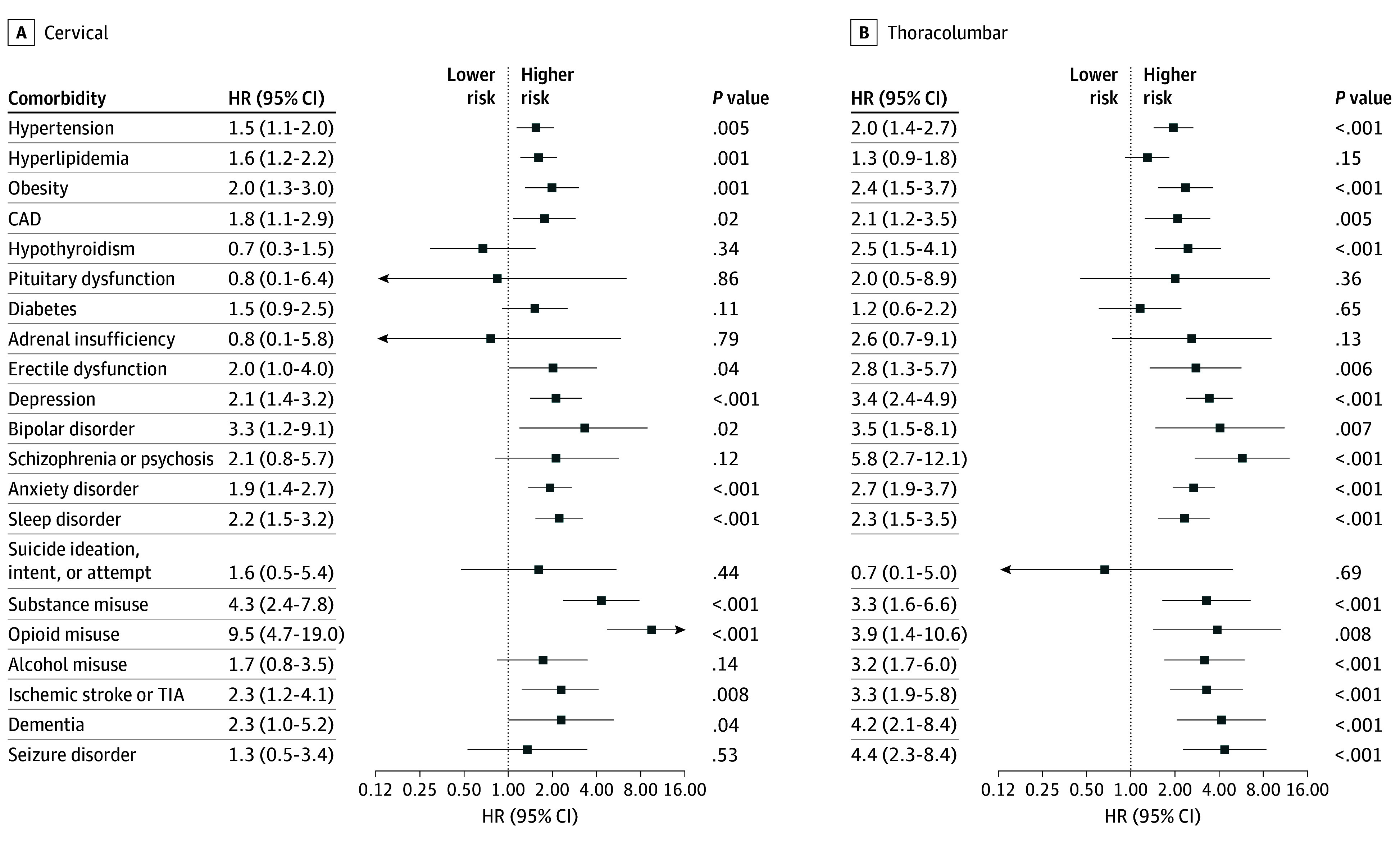
Risk of Multisystem Comorbidities After Traumatic Spinal Cord Injury, Stratified by Injury Location in the Mass General Brigham Cohort CAD indicates coronary artery disease; HR, hazard ratio; TIA, transient ischemic attack.

We found that the risk of long-term comorbidities persisted across different sites of spinal injuries in both cohorts. Cervical TSCI and thoracolumbar TSCI were associated with increased risk of hypertension in both cohorts (MGB, cervical: HR, 1.5; 95% CI, 1.1-2.0; MGB, thoracolumbar: HR, 2.0; 95% CI, 1.4-2.7; UC, cervical: HR, 1.5; 95% CI, 1.1-1.9; UC, thoracolumbar: HR, 1.4; 95% CI, 1.1-1.8). In addition, TSCI was associated with increased risk of obesity regardless of the injury site in both cohorts (Figure 4; eTables 10-11 in Supplement 1). An association of TSCI with coronary artery disease was found for both spinal injury levels in MGB but only for cervical injury in UC cohort (eTable 11 in Supplement 1). Among neurological disorders, only thoracolumbar TSCI was associated with dementia (HR, 4.2; 95% CI, 2.1-8.4) and with seizure (HR, 4.4; 95% CI, 2.3-8.4). Conversely in UC, only cervical TSCI was associated with increased risk of dementia (HR, 2.5; 95% CI, 1.3-5.1) and seizure (HR, 3.2; 95% CI, 1.8-5.7). Among endocrinological disorders, erectile dysfunction was associated with thoracolumbar and cervical TSCI in both cohorts. Finally, the site of TSCI did not affect the increased risk found between trauma and psychiatric diseases in either cohort (Figure 4; eTable 10-11 in Supplement 1).

### Mortality Risk With Spinal Cord Injury and Associated Multisystem Comorbidities

Patients with TSCI had higher risk of mortality compared with uninjured controls (142 of 1038 [14%] vs 295 of 3114 [10%]; *P* < .001) (eTable 12 in Supplement 1). In the MGB cohort, multiple post-TSCI diseases were also associated with increased mortality (eTable 13 in Supplement 1): hypertension (odds ratio [OR], 2.0 [95% CI, 1.2-3.5]), pituitary dysfunction (OR, 6.5 [95% CI, 1.1-33.2]), adrenal insufficiency (OR, 5.0 [95% CI, 1.04-20.2]), depression (OR, 2.9 [95% CI, 1.6-5.2]), substance misuse (OR, 4.0 [95% CI, 1.5-9.8]), seizures (OR, 6.4 [95% CI, 2.7-14.5]), and dementia (OR, 4.8 [95% CI, 2.0-11.6]).

### Time to Development of Comorbidities Following TSCI

Comorbidities across all systems emerged within a median (IQR) of 8.0 (3.7-12.6) years after TSCI in the MGB cohort. Generally, comorbidities occurred with shorter latency in the TSCI group compared with the uninjured group (eTable 14 in Supplement 1).

## Discussion

While the acute effects of TSCI are well characterized, its long-term systemic consequences remain understudied.^19^ In this multi-institutional study of tertiary trauma centers, we found that TSCI was associated with increased long-term risk of cardiovascular, neurologic, endocrine, and psychiatric comorbidities, even after excluding patients with baseline health conditions. These associations were robust across age groups and injury levels as well as after adjusting for comorbid TBI, suggesting that TSCI independently drives chronic multisystem disease. Our findings support the view that TSCI should be conceptualized not only as an acute injury but also as a chronic condition requiring long-term surveillance and management.

Consistent with prior research, we found that TSCI was associated with increased cardiovascular risk, including myocardial infarction, heart failure, atrial fibrillation and stroke.^12,14^ Notably, risk varied by injury level, with thoracic and lumbar injuries showing higher associations than cervical injuries. This may reflect differences in sympathetic nervous system disruption, particularly given the location of sympathetic outflow in the thoracic spine.^20,21^ In addition, we also identified increased risk of endocrine disorders, including adrenal insufficiency and pituitary dysfunction.^22^ Previous studies suggested altered testosterone and thyroid hormone levels after TSCI but were underpowered to detect rare conditions.^22,23^ Our larger sample enabled identification of these associations. Psychiatric and neurologic sequelae were common after TSCI. Depression and substance use were significantly elevated in our study, consistent with prior evidence.^24,25,26^ We also observed a significant association between TSCI and dementia and chronic seizure disorders, a finding previously described in one retrospective study and case reports.^13,27^ Our results confirm these associations in a US-based, racially diverse population, strengthening the evidence for an association between TSCI and subsequent epileptogenesis and neurodegenerative diseases.^28^

TBI has been reported to associate with long-term risks of multisystem comorbidities, and they could co-occur with TSCI, so it was essential to determine whether the associations observed in our study were independent of concurrent brain injury.^24,29,30^ Importantly, we confirmed that increased comorbidity burden following TSCI persisted even after adjusting for TBI and age, supporting the notion that trauma to the spinal cord alone can initiate systemic pathophysiological processes. Cross-validation in 2 large health systems further strengthens generalizability.

TSCI was also associated with elevated long-term mortality, particularly in patients developing cardiovascular, neurologic, and psychiatric comorbidities. While some results were not statistically significant due to sample size limitations, the overall pattern underscores the clinical relevance of these chronic sequelae. Further investigation is required regarding the directionality and causality of comorbidities and their association with long-term mortality.

Mechanistically, chronic neuroinflammation, autonomic dysregulation, and impaired cerebrovascular function may underlie these associations.^2,28,31,32^ In addition, behavioral changes—including physical inactivity, unhealthy diet, and social isolation—are more common after injury and are known contributors to cardiometabolic and neuropsychiatric disease.^33^ Additionally, emerging data suggest trauma may alter systemic metabolomics, gut microbiota, and immune function, promoting disease through independent biological pathways. Future longitudinal and mechanistic studies are needed to clarify these relationships.^21,34,35,36^

### Strengths and Limitations

Our study advances the spinal cord injury field by systematically evaluating a broad range of diseases, uses a long follow-up period, accounts for traumatic brain injury and for the first time examines whether the risk of comorbidities varies by the anatomical level of spinal cord injury.^13,23,24,29,30^ However, a few limitations are worth noting. This is a registry database study from tertiary academic health systems across Massachusetts and California and diagnoses are based on *ICD-9* and *ICD-10* codes. However, there is a high degree of concordance between manual medical record review and *ICD* coding.^17^ Exclusion of patients with preexisting conditions likely creates a selection bias toward a particularly healthy cohort, potentially limiting generalizability; however, this would likely bias our results toward the null hypothesis. We did not quantify TSCI severity or general trauma burden, which may influence comorbidity risk profiles. Some risk estimates may be influenced by small sample sizes. However, it is noteworthy that the direction of the findings remained consistent across cohorts, despite known regional, health care access patterns, and epidemiological differences. Wide confidence intervals were also noted in the mortality analysis, suggesting limited precision of some effect estimates. Additionally, although evaluated as independent outcomes, several comorbidities in our analysis are mechanistically interconnected and may influence one another’s development (eg, hypertension contributing to coronary artery disease). However, our study was not designed to assess potential mediation or causal pathways between these conditions.

## Conclusions

In this cohort study, TSCI was associated with elevated long-term risk for chronic multisystem disease and mortality, independent of TBI. These findings highlight the need for proactive, multidisciplinary long-term care strategies. Future studies should aim to elucidate underlying mechanisms and identify effective interventions to mitigate chronic disease burden in this vulnerable population.

## Notes

Supplement 1. **eTable 1. **ICD-9 and ICD-10 Codes Used for Determining Diagnosis **eTable 2**. Median Number of Encounters Before Diagnosis of Comorbidity **eTable 3.** Sensitivity Analysis—Hazard Ratios for Comorbidities Developing After TSCI in Patients Indexed Prior to 2015 **eTable 4. **Hazard Ratios for Comorbidities Developing After TSCI With Follow-Up Censored at 15 Years **eTable 5.** Hazard Ratio of Comorbidities Developing After TSCI Adjusted by TBI in the Mass General Brigham Cohort **eTable 6. **Hazard Ratio of Comorbidities Developing After TSCI Adjusted by TBI in the University of California Cohort **eTable 7. **Hazard Ratio of Comorbidities Developing After TSCI Stratified by Age in the Mass General Brigham Cohort **eTable 8**. Hazard Ratio of Comorbidities Developing After TSCI Stratified by Age in the University of California Cohort **eTable 9.** Interaction Analysis Between TSCI and Age in the MBG Cohort **eTable 10. **Hazard Ratio of Comorbidities Developing After TSCI Stratified by Spine Injury Location in the Mass General Brigham Cohort **eTable 11. **Hazard Ratio of Comorbidities Developing After TSCI Stratified by Spine Injury Location in the University of California Cohort **eTable 12**. Association Between TSCI and Mortality in MGB Cohort **eTable 13. **Logistic Regression Analysis of Associations Between Post–Spinal Cord Injury Comorbidities and Mortality in MGB Cohort—Adjusted for Age, Sex and Race **eTable 14. **Percentages and Hazard Ratios for Multisystem Comorbidities Developing After TSCI and Time to Development of Comorbidities After Index Date or TSCI Diagnosis, in TSCI and Control Groups in the MBG Cohort **eFigure 1.** Study Flow Chart for the Mass General Brigham (MGB) and the University of California (UC) Cohorts e**Figure 2.** Kaplan-Meier Curves of Risk of Multisystemic Comorbidities (Excluded From Figure 2) After TSCI in the Mass General Brigham Cohort **eFigure 3. **Kaplan-Meier Curves of Risk of the Multisystemic Comorbidities After TSCI in the University of California (UC) Cohort

Supplement 2.
Data Sharing Statement
