# Supplementary material for: Traumatic Spinal Cord Injury and Subsequent Risk of Developing Chronic Cardiovascular, Neurologic, Psychiatric, and Endocrine Disorders
Source: JAMA Netw Open. 2025 Nov 4;8(11):e2541157. doi: 10.1001/jamanetworkopen.2025.41157 (PMC12587198; doi:10.1001/jamanetworkopen.2025.41157)
Supplement: Supplement 1. — eTable 1. ICD-9 and ICD-10 Codes Used for Determining Diagnosis eTable 2. Median Number of Encounters Before Diagnosis of Comorbidity eTable 3. Sensitivity Analysis—Hazard Ratios for Comorbidities Developing After TSCI in Patients Indexed Prior to 2015 eTable 4. Hazard Ratios for Comorbidities Developing After TSCI With Follow-Up Censored at 15 Years eTable 5. Hazard Ratio of Comorbidities Developing After TSCI Adjusted by TBI in the Mass General Brigham Cohort eTable 6. Hazard Ratio of Comorbidities Developing After TSCI Adjusted by TBI in the University of California Cohort eTable 7. Hazard Ratio of Comorbidities Developing After TSCI Stratified by Age in the Mass General Brigham Cohort eTable 8. Hazard Ratio of Comorbidities Developing After TSCI Stratified by Age in the University of California Cohort eTable 9. Interaction Analysis Between TSCI and Age in the MBG Cohort eTable 10. Hazard Ratio of Comorbidities Developing After TSCI Stratified by Spine Injury Location in the Mass General Brigham Cohort eTable 11. Hazard Ratio of Comorbidities Developing After TSCI Stratified by Spine Injury Location in the University of California Cohort eTable 12. Association Between TSCI and Mortality in MGB Cohort eTable 13. Logistic Regression Analysis of Associations Between Post–Spinal Cord Injury Comorbidities and Mortality in MGB Cohort—Adjusted for Age, Sex and Race eTable 14. Percentages and Hazard Ratios for Multisystem Comorbidities Developing After TSCI and Time to Development of Comorbidities After Index Date or TSCI Diagnosis, in TSCI and Control Groups in the MBG Cohort eFigure 1. Study Flow Chart for the Mass General Brigham (MGB) and the University of California (UC) Cohorts eFigure 2. Kaplan-Meier Curves of Risk of Multisystemic Comorbidities (Excluded From Figure 2) After TSCI in the Mass General Brigham Cohort eFigure 3. Kaplan-Meier Curves of Risk of the Multisystemic Comorbidities After TSCI in the University of California (UC) Cohort [file jamanetwopen-e2541157-s001.pdf]

Supplemental Online Content

Mashlah A, Marini S, Mills H, et al. Traumatic Spinal Cord Injury and Subsequent Risk of Developing Chronic Disorders. *JAMA Netw Open.* 2025;8(11):e2541157.
doi:10.1001/jamanetworkopen.2025.41157

eTable 1. ICD-9 and ICD-10 Codes Used for Determining Diagnosis

eTable 2 . Median Number of Encounters Before Diagnosis of Comorbidity

eTable 3. Sensitivity Analysis—Hazard Ratios for Comorbidities Developing After TSCI in Patients Indexed Prior to 2015

eTable 4. Hazard Ratios for Comorbidities Developing After TSCI With Follow-Up Censored at 15 Years

eTable 5. Hazard Ratio of Comorbidities Developing After TSCI Adjusted by TBI in the Mass General Brigham Cohort

eTable 6. Hazard Ratio of Comorbidities Developing After TSCI Adjusted by TBI in the University of California Cohort

eTable 7. Hazard Ratio of Comorbidities Developing After TSCI Stratified by Age in the Mass General Brigham Cohort

eTable 8 . Hazard Ratio of Comorbidities Developing After TSCI Stratified by Age in the University of California Cohort

eTable 9. Interaction Analysis Between TSCI and Age in the MBG Cohort

eTable 10. Hazard Ratio of Comorbidities Developing After TSCI Stratified by Spine Injury Location in the Mass General Brigham Cohort

eTable 11. Hazard Ratio of Comorbidities Developing After TSCI Stratified by Spine Injury Location in the University of California Cohort

eTable 12 . Association Between TSCI and Mortality in MGB Cohort

eTable 13. Logistic Regression Analysis of Associations Between Post–Spinal Cord Injury Comorbidities and Mortality in MGB Cohort—Adjusted for Age, Sex and Race

eTable 14. Percentages and Hazard Ratios for Multisystem Comorbidities Developing After TSCI and Time to Development of Comorbidities After Index Date or TSCI Diagnosis, in TSCI and Control Groups in the MBG Cohort

eFigure 1. Study Flow Chart for the Mass General Brigham (MGB) and the University of California (UC) Cohorts

e**Figure 2.** Kaplan-Meier Curves of Risk of Multisystemic Comorbidities (Excluded From Figure 2) After TSCI in the Mass General Brigham Cohort

eFigure 3. Kaplan-Meier Curves of Risk of the Multisystemic Comorbidities After TSCI in the University of California (UC) Cohort

© 2025 Mashlah A et al. *JAMA Network Open*.

This supplemental material has been provided by the authors to give readers additional information about their work.

© 2025 Mashlah A et al. *JAMA Network Open*.

eTable 1. ICD-9 and ICD-10 Codes Used for Determining Diagnosis

Disorder	ICD-9	ICD-10
**Cardiovascular Disorders**		
Hypertension	401, 402, 403, 404, 405	I10, I11, I12, I13, I15, I16
Hyperlipidemia	272	E78
Obesity	278	E66, Z68.25-Z68.44
Coronary artery disease	410, 411, 412, 413, 414	I21, I22, I23, I24, I25
**Endocrine disorders**		
Hypothyroidism	244	E03
Pituitary dysfunction	253	E23
Diabetes mellitus	250, 790.29, 790.21	E08, E09, E11, E13
Adrenal insufficiency	255.4-255.9	E27.0-E27.7
Erectile dysfunction	607.84	N52, F52.21
**Psychiatric disorders**		
Depression	296.2, 296.3, 296.8, 300.4, 311	F32, F33
Bipolar disorder	296.4, 296.5, 296.6, 296.7	F25, F31
Schizophrenia/ psychosis	295, 296.89, 296.9, 298	F06, F20, F22, F23, F28, F29
Anxiety disorder	300, 300.2, 308, 309	F41, F40.8, F40.9, F43.2
Sleep disorder	307.4, 327, 780.5, 347	F51, G47
Suicide ideation/intent/attempt	E950 - E958, V62.84	R45. 851, X83, T40, Z91.5
Substance misuse	304.1-304.6, 304.8-304.9, 305.2, 305.3, 305.4, 305.6-305.9	F12, F13, F14, F15, F16, F18, F19
Opioid misuse	304.0, 304.7, 305.5	F11
Alcohol misuse	303, 305.0	F10
**Neurological disorders**		
Ischemic stroke/TIA	433, 434, 435, 436, 437	I63, I65, I66, I67
Dementia	290.0-290.4, 290.8, 290.9, 294.1, 294.2, 331	F01, F02, F03, G30, G31
Seizure disorder	780.33, 780.39, 345	R56.1, R56.9, G40
**Spinal Cord Injuries:**		
Cervical	806.0, 806.1, 952.0	S14.0, S14.1, S14.2
Thoracolumbar	806.2, 806.3, 806.4, 806.5, 806.6, 806.7, 952.1	S24.0, S24.1, S24.2, S34.0-S34.3
Unspecified	806.8, 806.9, 952.2-952.9	

© 2025 Mashlah A et al. *JAMA Network Open*.

eTable 2. Median Number of Encounters Before Diagnosis of Comorbidity

Diagnosis	Uninjured - Median number of encounters before diagnosis	SCI - Median number of encounters before diagnosis
**Hypertension**	5.00 (2.00-10.00)	7.00 (3.00-17.00)*
**Hyperlipidemia**	6.00 (3.00-11.00)	8.00 (3.00-17.00)*
**Obesity**	8.00 (3.00-18.50)	11.00 (6.00-22.00)*
**Coronary artery disease**	7.00 (3.00-17.75)	9.00 (4.00-26.00)*
**Hypothyroidism**	7.00 (4.00-13.00)	7.00 (4.00-14.25)
**Pituitary dysfunction**	9.00 (4.00-30.00)	16.50 (6.75-19.75)
**Diabetes mellitus**	4.00 (2.00-12.50)	7.00 (3.00-15.50)*
**Adrenal insufficiency**	6.00 (3.50-17.50)	11.00 (4.00-16.00)
**Erectile dysfunction**	12.00 (4.00-21.00)	8.00 (5.00-17.00)
**Depression**	7.00 (3.00-13.75)	7.00 (4.00-15.00)
**Bipolar disorder**	5.00 (2.00-13.25)	5.00 (4.00-10.00)
**Schizophrenia/psychosis**	5.00 (2.75-14.00)	8.00 (3.50-15.50)
**Anxiety disorder**	8.00 (4.00-16.00)	9.00 (4.00-18.00)
**Sleep disorder**	7.50 (4.00-16.00)	12.00 (6.00-25.75)*
**Suicide ideation/intent/attempt**	4.00 (1.00-12.50)	17.50 (4.00-33.25)
**Substance misuse**	5.00 (2.00-8.00)	6.00 (2.25-14.50)
**Opioid misuse**	4.50 (2.00-12.50)	7.00 (4.00-15.00)
**Alcohol misuse**	4.00 (1.50-9.00)	6.00 (2.00-16.50)
**Ischemic stroke/TIA**	7.00 (3.00-13.50)	12.50 (4.00-24.00)*
**Dementia**	8.00 (5.00-21.50)	13.00 (4.00-22.00)
**Seizure disorder**	5.00 (2.75-9.25)	9.50 (3.00-30.50)

Significance denoted by * < 0.05
Reference= Uninjured group, Wilcoxon Rank-Sum Test

© 2025 Mashlah A et al. *JAMA Network Open*.

eTable 3. Sensitivity Analysis—Hazard Ratios for Comorbidities Developing After TSCI in Patients Indexed Prior to 2015

Diagnosis	SCI N = 782	Control N=2346	HR (95% CI)
**Hypertension**	322 (14%)	157 (20%)	1.70 (1.40-2.07)*
**Hyperlipidemia**	109 (4.6%)	57 (7.3%)	1.64 (1.34-1.99)*
**Obesity**	298 (13%)	148 (19%)	1.89 (1.43-2.50)*
**Coronary artery disease**	132 (5.6%)	81 (10%)	1.87 (1.35-2.59)*
**Hypothyroidism**	80 (3.4%)	47 (6.0%)	1.81 (1.26-2.60)*
**Pituitary dysfunction**	12 (0.5%)	8 (1.0%)	1.97 (0.80-4.85)
**Diabetes mellitus**	103 (4.4%)	49 (6.3%)	1.48 (1.05-2.08)*
**Adrenal insufficiency**	11 (0.5%)	11 (1.4%)	3.14 (1.36-7.26)*
**Erectile dysfunction**	36 (1.5%)	20 (2.6%)	1.85 (1.06-3.24)*
**Depression**	137 (5.8%)	119 (15%)	2.85 (2.23-3.66)*
**Bipolar disorder**	12 (0.5%)	16 (2.0%)	4.10 (1.89-8.86)*
**Schizophrenia/psychosis**	29 (1.2%)	30 (3.8%)	3.09 (1.85-5.15)*
**Anxiety disorder**	200 (8.5%)	147 (19%)	2.45 (1.97-3.04)*
**Sleep disorder**	135 (5.8%)	96 (12%)	2.35 (1.80-3.06)*
**Suicide ideation/intent/attempt**	20 (0.9%)	14 (1.8%)	2.16 (1.08-4.34)*
**Substance misuse**	37 (1.6%)	38 (4.9%)	3.26 (2.06-5.16)*
**Opioid misuse**	17 (0.7%)	36 (4.6%)	6.42 (3.60-11.47)*
**Alcohol misuse**	49 (2.1%)	37 (4.7%)	2.44 (1.58-3.77)*
**Ischemic stroke/TIA**	68 (2.9%)	42 (5.4%)	2.18 (1.47-3.22)*
**Dementia**	50 (2.1%)	28 (3.6%)	2.00 (1.25-3.21)*
**Seizure disorder**	27 (1.2%)	31 (4.0%)	3.59 (2.13-6.07)*
Adjusted for age, sex and race<br>Significance denoted by p-value <0.05<br>Reference = Uninjured group			

© 2025 Mashlah A et al. *JAMA Network Open*.

eTable 4. Hazard Ratios for Comorbidities Developing After TSCI With Follow-Up Censored at 15 Years

Diagnosis	SCI<br>N = 1038	Control<br>N=3114	HR (95% CI)
**Hypertension**	315 (10%)	149 (14%)	1.60 (1.32-1.95)*
**Hyperlipidemia**	93 (3.0%)	47 (4.5%)	1.48 (1.21-1.81)*
**Obesity**	312 (10%)	142 (14%)	2.05 (1.53-2.76)*
**Coronary artery disease**	108 (3.5%)	75 (7.2%)	1.64 (1.15-2.34)*
**Hypothyroidism**	89 (2.9%)	42 (4.0%)	1.42 (0.98-2.05)
**Pituitary dysfunction**	9 (0.3%)	7 (0.7%)	2.34 (0.87-6.29)
**Diabetes mellitus**	93 (3.0%)	45 (4.3%)	1.47 (1.03-2.10)*
**Adrenal insufficiency**	12 (0.4%)	11 (1.1%)	2.94 (1.29-6.70)*
**Erectile dysfunction**	36 (1.2%)	22 (2.1%)	2.00 (1.17-3.41)*
**Depression**	129 (4.1%)	111 (11%)	2.73 (2.11-3.52)*
**Bipolar disorder**	11 (0.4%)	15 (1.4%)	3.87 (1.77-8.46)*
**Schizophrenia/psychosis**	20 (0.6%)	26 (2.5%)	3.97 (2.21-7.13)*
**Anxiety disorder**	197 (6.3%)	139 (13%)	2.21 (1.77-2.74)*
**Sleep disorder**	136 (4.4%)	77 (7.4%)	1.79 (1.35-2.37)*
**Suicide ideation/intent/attempt**	17 (0.5%)	7 (0.7%)	1.28 (0.53-3.09)
**Substance misuse**	34 (1.1%)	33 (3.2%)	3.03 (1.87-4.90)*
**Opioid misuse**	14 (0.4%)	29 (2.8%)	6.15 (3.24-11.66)*
**Alcohol misuse**	47 (1.5%)	30 (2.9%)	2.00 (1.26-3.17)*
**Ischemic stroke/TIA**	50 (1.6%)	40 (3.9%)	2.64 (1.74-4.01)*
**Dementia**	26 (0.8%)	21 (2.0%)	2.60 (1.46-4.63)*
**Seizure disorder**	29 (0.9%)	28 (2.7%)	2.95 (1.75-4.97)*
Adjusted for age, sex and race<br>Significance denoted by p-value <0.05<br>Reference = Uninjured group			

© 2025 Mashlah A et al. *JAMA Network Open*.

eTable 5. Hazard Ratio of Comorbidities Developing After TSCI Adjusted by TBI in the Mass General Brigham Cohort

Diagnosis	HR (95% CI)	p-value
**Hypertension**	2.45 (2.03 - 2.96)*	<0.001
**Hyperlipidemia**	2.41 (1.99 - 2.93)*	<0.001
**Obesity**	2.91 (2.20 - 3.85)*	<0.001
**Coronary artery disease**	2.70 (1.96 - 3.72)*	<0.001
**Hypothyroidism**	2.12 (1.48 - 3.04)*	<0.001
**Pituitary dysfunction**	1.99 (0.79 - 5.03)	0.147
**Diabetes mellitus**	2.19 (1.55 - 3.09)*	<0.001
**Adrenal insufficiency**	3.79 (1.68 - 8.53)*	0.001
**Erectile dysfunction**	2.89 (1.73 - 4.83)*	<0.001
**Depression**	4.01 (3.11 - 5.16)*	<0.001
**Bipolar disorder**	6.14 (3.01 - 12.50)*	<0.001
**Schizophrenia/psychosis**	5.51 (3.10 - 9.81)*	<0.001
**Anxiety disorder**	3.52 (2.84 - 4.36)*	<0.001
**Sleep disorder**	3.03 (2.34 - 3.91)*	<0.001
**Suicide ideation/intent/attempt**	2.61 (1.26 - 5.38)	0.010
**Substance misuse**	4.41 (2.78 - 7.01)*	<0.001
**Opioid misuse**	9.66 (5.30 - 17.62)*	<0.001
**Alcohol misuse**	3.04 (1.95 - 4.73)*	<0.001
**Ischemic stroke/TIA**	3.12 (2.09 - 4.65)*	<0.001
**Dementia**	3.67 (2.18 - 6.15)*	<0.001
**Seizure disorder**	3.63 (2.20 - 5.98)*	<0.001
Adjusted for age, sex, race and concurrent TBI<br>Significance denoted by * if p< 0.002 (Bonferroni Adjustment)<br>Reference = Uninjured group		

© 2025 Mashlah A et al. *JAMA Network Open*.

eTable 6. Hazard Ratio of Comorbidities Developing After TSCI Adjusted by TBI in the University of California Cohort

Diagnosis	HR (95% CI)	p-value
**Hypertension**	1.57 (1.34-1.84)*	<0.001
**Hyperlipidemia**	1.16 (0.98-1.38)	0.083
**Obesity**	1.80 (1.47-2.20)*	<0.001
**Coronary artery disease**	1.81 (1.35-2.43)*	<0.001
**Hypothyroidism**	1.23 (0.91-1.67)	0.174
**Pituitary dysfunction**	5.31 (2.22-12.69)*	<0.001
**Diabetes mellitus**	1.25 (1.03-1.53)	0.028
**Adrenal insufficiency**	5.62 (2.73-11.58)*	<0.001
**Erectile dysfunction**	3.00 (2.23-4.03)*	<0.001
**Depression**	2.17 (1.8-2.6)*	<0.001
**Bipolar disorder**	0.61 (0.29-1.31)	0.210
**Schizophrenia psychosis**	2.12 (1.41-3.19)*	<0.001
**Anxiety disorder**	2.26 (1.91-2.67)*	<0.001
**Sleep disorder**	1.98 (1.6-2.4)*	<0.001
**Suicide ideation intent attempt**	4.45 (2.76-7.20)*	<0.001
**Substance misuse**	2.30 (1.70-3.10)*	<0.001
**Opioid misuse**	10.13 (6.39-16.1)*	<0.001
**Alcohol misuse**	1.39 (0.90-2.13)	0.136
**Ischemic stroke TIA**	1.60 (1.08-2.39)	0.019
**Dementia**	2.37 (1.51-3.72)*	<0.001
**Seizure disorder**	2.72 (1.78-4.16)*	<0.001

Adjusted for age, sex, race and concurrent TBI
Significance denoted by * if p< 0.002 (Bonferroni Adjustment)
Reference = Uninjured group

© 2025 Mashlah A et al. *JAMA Network Open*.

eTable 7. Hazard Ratio of Comorbidities Developing After TSCI Stratified by Age in the Mass General Brigham Cohort

**Diagnosis**	**Age Group**	**HR (CI 95%)**	**p-value**
Hypertension	18-45	1.54 (1.14 - 2.08)	0.005
Hypertension	45-65	1.46 (1.10 - 1.94)	0.010
Hypertension	65	1.82 (1.24 - 2.67)	0.002
Hyperlipidemia	18-45	1.29 (0.96 - 1.73)	0.091
Hyperlipidemia	45-65	1.65 (1.25 - 2.17)	<0.001
Hyperlipidemia	65	1.81 (1.11 - 2.93)	0.017
Obesity	18-45	2.01 (1.44 - 2.80)	<0.001
Obesity	45-65	2.29 (1.41 - 3.70)	0.001
Obesity	65	1.01 (0.28 - 3.60)	0.994
Coronary artery disease	18-45	1.66 (0.87 - 3.20)	0.127
Coronary artery disease	45-65	1.72 (1.11 - 2.67)	0.015
Coronary artery disease	65	1.70 (0.93 - 3.12)	0.084
Hypothyroidism	18-45	1.26 (0.72 - 2.18)	0.418
Hypothyroidism	45-65	1.66 (0.96 - 2.89)	0.069
Hypothyroidism	65	2.01 (0.94 - 4.29)	0.073
Pituitary dysfunction	18-45	1.63 (0.48 - 5.55)	0.438
Pituitary dysfunction	45-65	1.63 (0.39 - 6.81)	0.506
Pituitary dysfunction	65	3.72 (0.23 - 59.42)	0.353
Diabetes Mellitus	18-45	1.71 (1.03 - 2.81)	0.037
Diabetes Mellitus	45-65	1.91 (1.14 - 3.20)	0.014
Diabetes Mellitus	65	0.53 (0.19 - 1.53)	0.241
Adrenal insufficiency	18-45	2.25 (0.50 - 10.07)	0.288
Adrenal insufficiency	45-65	2.78 (0.90 - 8.62)	0.077
Adrenal insufficiency	65	1.97 (0.36 - 10.79)	0.434
Erectile dysfunction	18-45	1.69 (0.84 - 3.43)	0.144
Erectile dysfunction	45-65	1.66 (0.81 - 3.39)	0.167
Erectile dysfunction	65	1.27 (0.13 - 12.16)	0.839
Depression	18-45	2.59 (1.90 - 3.53)	<0.001
Depression	45-65	3.00 (1.93 - 4.66)	<0.001
Depression	65	2.73 (1.34 - 5.58)	0.006
Bipolar disorder	18-45	4.95 (2.34 - 10.49)	<0.001
Bipolar disorder	45-65	NA	NA
Bipolar disorder	65	NA	NA
Schizophrenia/psychosis	18-45	5.13 (2.58 - 10.18)	<0.001
Schizophrenia/psychosis	45-65	3.21 (0.98 - 10.54)	0.054
Schizophrenia/psychosis	65	2.62 (0.62 - 11.03)	0.189
Anxiety disorder	18-45	2.28 (1.76 - 2.95)	<0.001
Anxiety disorder	45-65	2.37 (1.64 - 3.42)	<0.001
Anxiety disorder	65	2.52 (1.22 - 5.20)	0.012
Sleep disorder	18-45	1.93 (1.38 - 2.72)	<0.001
Sleep disorder	45-65	1.81 (1.22 - 2.69)	0.003

© 2025 Mashlah A et al. *JAMA Network Open*.

Diagnosis	Age Group	HR (CI 95%)	p-value
Sleep disorder	65	2.21 (0.98 - 5.01)	0.057
Suicide ideation/intent/attempt	18-45	3.03 (1.36 - 6.75)	0.007
Suicide ideation/intent/attempt	45-65	1.09 (0.21 - 5.64)	0.917
Suicide ideation/intent/attempt	65	NA	NA
Substance misuse	18-45	3.17 (1.96 - 5.14)	<0.001
Substance misuse	45-65	2.96 (1.03 - 8.48)	0.043
Substance misuse	65	NA	NA
Opioid misuse	18-45	5.20 (2.67 - 10.11)	<0.001
Opioid misuse	45-65	14.84 (3.29 - 67.00)	<0.001
Opioid misuse	65	13.93 (1.42 - 136.42)	0.024
Alcohol misuse	18-45	2.23 (1.36 - 3.66)	0.002
Alcohol misuse	45-65	2.28 (1.07 - 4.89)	0.034
Alcohol misuse	65	NA	NA
Ischemic stroke/TIA	18-45	2.80 (1.32 - 5.96)	0.007
Ischemic stroke/TIA	45-65	1.92 (1.07 - 3.45)	0.030
Ischemic stroke/TIA	65	2.94 (1.48 - 5.84)	0.002
Dementia	18-45	2.29 (0.61 - 8.53)	0.217
Dementia	45-65	4.05 (1.67 - 9.84)	0.002
Dementia	65	3.12 (1.52 - 6.39)	0.002
Seizure disorder	18-45	2.88 (1.45 - 5.70)	0.002
Seizure disorder	45-65	3.40 (1.67 - 6.91)	0.001
Seizure disorder	65	0.75 (0.09 - 6.41)	0.792
Adjusted for age, sex and race<br>Reference = Uninjured group			

© 2025 Mashlah A et al. *JAMA Network Open*.

eTable 8. Hazard Ratio of Comorbidities Developing After TSCI Stratified by Age in the University of California Cohort

Age Group	Diagnosis	HR	p-value
18-45	Hypertension	2.07 (1.55 - 2.77)	<0.001
45-65	Hypertension	1.25 (0.99 - 1.57)	0.063
65+	Hypertension	1.56 (1.15 - 2.12)	0.004
18-45	Hyperlipidemia	1.15 (0.83 - 1.60)	0.387
45-65	Hyperlipidemia	1.01 (0.80 - 1.29)	0.914
65+	Hyperlipidemia	1.37 (0.97 - 1.94)	0.070
18-45	Obesity	1.63 (1.22 - 2.17)	0.001
45-65	Obesity	1.55 (1.12 - 2.13)	0.008
65+	Obesity	3.37 (1.98 - 5.72)	<0.001
18-45	Coronary artery disease	3.19 (1.47 - 6.90)	0.003
45-65	Coronary artery disease	1.52 (1.02 - 2.27)	0.042
65+	Coronary artery disease	1.72 (1.06 - 2.79)	0.027
18-45	Hypothyroidism	1.08 (0.60 - 1.95)	0.787
45-65	Hypothyroidism	1.39 (0.91 - 2.13)	0.132
65+	Hypothyroidism	1.14 (0.64 - 2.05)	0.653
18-45	Pituitary dysfunction	3.79 (1.11 - 12.88)	0.033
45-65	Pituitary dysfunction	20.52 (2.85 - 147.52)	0.003
65+	Pituitary dysfunction	3.60 (0.46 - 28.22)	0.222
18-45	Diabetes mellitus	1.27 (0.87 - 1.84)	0.215
45-65	Diabetes mellitus	1.12 (0.85 - 1.48)	0.432
65+	Diabetes mellitus	1.29 (0.85 - 1.96)	0.238
18-45	Adrenal insufficiency	692.74 (385.77 - 1243.97)	<0.001
45-65	Adrenal insufficiency	3.39 (1.18 - 9.73)	0.023
65+	Adrenal insufficiency	2.89 (0.59 - 14.26)	0.192
18-45	Erectile dysfunction	5.90 (3.71 - 9.38)	<0.001
45-65	Erectile dysfunction	1.80 (1.14 - 2.83)	0.011
65+	Erectile dysfunction	0.89 (0.31 - 2.56)	0.833
18-45	Depression	2.25 (1.76 - 2.88)	<0.001
45-65	Depression	2.43 (1.79 - 3.28)	<0.001
65+	Depression	1.64 (1.01 - 2.65)	0.046
18-45	Bipolar disorder	0.72 (0.27 - 1.89)	0.506
45-65	Bipolar disorder	0.44 (0.13 - 1.55)	0.202
65+	Bipolar disorder	0.01 (0.00 - 0.06)	0.000
18-45	Schizophrenia psychosis	1.82 (0.95 - 3.48)	0.072
45-65	Schizophrenia psychosis	2.73 (1.42 - 5.27)	0.003
65+	Schizophrenia psychosis	1.76 (0.75 - 4.16)	0.195
18-45	Anxiety disorder	2.18 (1.74 - 2.73)	<0.001
45-65	Anxiety disorder	2.47 (1.86 - 3.28)	<0.001
65+	Anxiety disorder	2.68 (1.70 - 4.25)	<0.001
18-45	Sleep disorder	2.04 (1.53 - 2.72)	<0.001
45-65	Sleep disorder	1.71 (1.28 - 2.29)	<0.001

© 2025 Mashlah A et al. *JAMA Network Open*.

65+	Sleep disorder	2.07 (1.35 - 3.19)	0.001
18-45	Suicide ideation intent attempt	4.69 (2.43 - 9.03)	0.000
45-65	Suicide ideation intent attempt	3.93 (1.79 - 8.61)	0.001
65+	Suicide ideation intent attempt	2.40 (0.53 - 10.94)	0.257
18-45	Substance misuse	2.48 (1.71 - 3.61)	<0.001
45-65	Substance misuse	1.68 (1.00 - 2.80)	0.048
65+	Substance misuse	5.39 (1.48 - 19.60)	0.011
18-45	Opioid misuse	7.65 (4.16 - 14.07)	<0.001
45-65	Opioid misuse	13.52 (6.29 - 29.04)	<0.001
65+	Opioid misuse	11.90 (2.78 - 51.03)	0.001
18-45	Alcohol misuse	1.28 (0.74 - 2.23)	0.380
45-65	Alcohol misuse	1.39 (0.69 - 2.83)	0.356
65+	Alcohol misuse	4.83 (1.16 - 20.04)	0.030
18-45	Ischemic stroke TIA	2.55 (1.12 - 5.78)	0.025
45-65	Ischemic stroke TIA	1.73 (0.92 - 3.25)	0.089
65+	Ischemic stroke TIA	1.16 (0.61 - 2.22)	0.653
18-45	Dementia	1.53 (0.28 - 8.47)	0.627
45-65	Dementia	5.45 (1.89 - 15.73)	0.002
65+	Dementia	1.99 (1.14 - 3.48)	0.015
18-45	Seizure disorder	2.67 (1.50 - 4.76)	0.001
45-65	Seizure disorder	3.14 (1.60 - 6.17)	0.001
65+	Seizure disorder	1.20 (0.23 - 6.09)	0.830
Adjusted for age, sex and race<br>Reference = Uninjured group			

© 2025 Mashlah A et al. *JAMA Network Open*.

Comorbidity	Interaction: Age Group 45-65 with SCI*	Interaction: Age Group > 65 with SCI*
Hypertension	0.99 (95% CI: 0.65 to 1.50), p=0.9578	1.26 (95% CI: 0.78 to 2.05), p=0.3453
Hyperlipidemia	1.29 (95% CI: 0.86 to 1.93), p=0.2186	1.41 (95% CI: 0.80 to 2.47), p=0.2324
Obesity	1.17 (95% CI: 0.65 to 2.09), p=0.6009	0.56 (95% CI: 0.15 to 2.09), p=0.3863
CAD	1.14 (95% CI: 0.52 to 2.46), p=0.7478	1.09 (95% CI: 0.45 to 2.63), p=0.8422
Hypothyroidism	1.25 (95% CI: 0.58 to 2.72), p=0.5694	1.57 (95% CI: 0.62 to 4.02), p=0.3439
Pituitary dysfunction	0.94 (95% CI: 0.14 to 6.22), p=0.9519	2.42 (95% CI: 0.12 to 50.32), p=0.5671
Diabetes Mellitus	1.14 (95% CI: 0.56 to 2.33), p=0.7106	0.34 (95% CI: 0.11 to 1.10), p=0.0714
Adrenal insufficiency	1.23 (95% CI: 0.19 to 8.02), p=0.8299	0.87 (95% CI: 0.09 to 8.35), p=0.9022
Erectile dysfunction	0.98 (95% CI: 0.36 to 2.68), p=0.9740	0.81 (95% CI: 0.08 to 8.64), p=0.8595
Depression	1.09 (95% CI: 0.64 to 1.84), p=0.7630	1.09 (95% CI: 0.50 to 2.38), p=0.8217
Bipolar disorder	NA	NA
Schizophrenia/psychosis	0.62 (95% CI: 0.16 to 2.45), p=0.4973	0.47 (95% CI: 0.10 to 2.28), p=0.3472
Anxiety disorder	1.02 (95% CI: 0.65 to 1.59), p=0.9366	1.12 (95% CI: 0.52 to 2.42), p=0.7679
Sleep disorder	0.98 (95% CI: 0.58 to 1.66), p=0.9498	1.29 (95% CI: 0.53 to 3.13), p=0.5684
Suicide ideation	0.33 (95% CI: 0.06 to 1.95), p=0.2205	NA
Substance misuse	0.89 (95% CI: 0.28 to 2.83), p=0.8494	NA
Opioid misuse	2.99 (95% CI: 0.58 to 15.53), p=0.1921	2.35 (95% CI: 0.22 to 24.82), p=0.4787
Alcohol misuse	1.04 (95% CI: 0.42 to 2.56), p=0.9404	NA
Ischemic stroke/TIA	0.64 (95% CI: 0.25 to 1.65), p=0.3532	1.03 (95% CI: 0.37 to 2.84), p=0.9541
Dementia	1.09 (95% CI: 0.23 to 5.09), p=0.9122	1.25 (95% CI: 0.28 to 5.57), p=0.7730
Seizure disorder	1.24 (95% CI: 0.47 to 3.29), p=0.6677	0.28 (95% CI: 0.03 to 2.70), p=0.2736

© 2025 Mashlah A et al. *JAMA Network Open*.

Reference= 18-45 age group with TSCI

eTable 9. Interaction Analysis Between TSCI and Age in the MBG Cohort

© 2025 Mashlah A et al. *JAMA Network Open*.

eTable 10. Hazard Ratio of Comorbidities Developing After TSCI Stratified by Spine Injury Location in the Mass General Brigham Cohort

Diagnosis	SCI Status	HR (CI 95%)	p-value
**Hypertension**	Cervical	1.53 (1.14 - 2.05)	0.005
**Hypertension**	Thoracolumbar	1.95 (1.43 - 2.66)	<0.001
**Hyperlipidemia**	Cervical	1.61 (1.21 - 2.15)	0.001
**Hyperlipidemia**	Thoracolumbar	1.29 (0.91 - 1.83)	0.150
**Obesity**	Cervical	1.98 (1.30 - 3.03)	0.001
**Obesity**	Thoracolumbar	2.37 (1.53 - 3.65)	<0.001
**Coronary artery disease**	Cervical	1.76 (1.08 - 2.87)	0.023
**Coronary artery disease**	Thoracolumbar	2.09 (1.25 - 3.48)	0.005
**Hypothyroidism**	Cervical	0.67 (0.29 - 1.53)	0.344
**Hypothyroidism**	Thoracolumbar	2.46 (1.47 - 4.12)	0.001
**Pituitary dysfunction**	Cervical	0.83 (0.11 - 6.39)	0.861
**Pituitary dysfunction**	Thoracolumbar	2.01 (0.45 - 8.91)	0.359
**Diabetes Mellitus**	Cervical	1.52 (0.91 - 2.54)	0.112
**Diabetes Mellitus**	Thoracolumbar	1.16 (0.61 - 2.22)	0.653
**Adrenal insufficiency**	Cervical	0.77 (0.10 - 5.84)	0.797
**Adrenal insufficiency**	Thoracolumbar	2.60 (0.74 - 9.11)	0.134
**Erectile dysfunction**	Cervical	2.02 (1.01 - 4.02)	0.046
**Erectile dysfunction**	Thoracolumbar	2.77 (1.35 - 5.69)	0.006
**Depression**	Cervical	2.11 (1.41 - 3.17)	<0.001
**Depression**	Thoracolumbar	3.43 (2.38 - 4.95)	<0.001
**Bipolar disorder**	Cervical	3.29 (1.19 - 9.12)	0.022
**Bipolar disorder**	Thoracolumbar	4.06 (1.47 - 11.21)	0.007
**Schizophrenia/psychosis**	Cervical	2.14 (0.81 - 5.65)	0.123
**Schizophrenia/psychosis**	Thoracolumbar	5.76 (2.74 - 12.12)	<0.001
**Anxiety disorder**	Cervical	1.92 (1.37 - 2.71)	<0.001
**Anxiety disorder**	Thoracolumbar	2.69 (1.93 - 3.74)	<0.001
**Sleep disorder**	Cervical	2.22 (1.53 - 3.22)	<0.001
**Sleep disorder**	Thoracolumbar	2.30 (1.53 - 3.46)	<0.001
**Suicide ideation/intent/attempt**	Cervical	1.61 (0.48 - 5.45)	0.445
**Suicide ideation/intent/attempt**	Thoracolumbar	0.66 (0.09 - 4.96)	0.691
**Substance misuse**	Cervical	4.30 (2.37 - 7.81)	<0.001
**Substance misuse**	Thoracolumbar	3.29 (1.65 - 6.60)	0.001
**Opioid misuse**	Cervical	9.48 (4.71 - 19.07)	<0.001
**Opioid misuse**	Thoracolumbar	3.89 (1.43 - 10.56)	0.008
**Alcohol misuse**	Cervical	1.70 (0.84 - 3.47)	0.143
**Alcohol misuse**	Thoracolumbar	3.18 (1.70 - 5.98)	<0.001
**Ischemic stroke/TIA**	Cervical	2.26 (1.24 - 4.12)	0.008
**Ischemic stroke/TIA**	Thoracolumbar	3.29 (1.86 - 5.82)	<0.001
**Dementia**	Cervical	2.29 (1.00 - 5.23)	0.049
**Dementia**	Thoracolumbar	4.16 (2.06 - 8.37)	<0.001
**Seizure disorder**	Cervical	1.35 (0.53 - 3.45)	0.532
**Seizure disorder**	Thoracolumbar	4.37 (2.27 - 8.42)	<0.001
Adjusted for age, sex and race			

© 2025 Mashlah A et al. *JAMA Network Open*.

Reference = Uninjured group

© 2025 Mashlah A et al. *JAMA Network Open*.

Diagnosis	Location	HR (95% CI)	p-value
**Hypertension**	Cervical	1.46 (1.14 - 1.87)	0.003
**Hypertension**	Thoracolumbar	1.37 (1.07 - 1.76)	0.013
**Hyperlipidemia**	Cervical	1.11 (0.85 - 1.45)	0.442
**Hyperlipidemia**	Thoracolumbar	1.11 (0.85 - 1.46)	0.426
**Obesity**	Cervical	1.82 (1.34 - 2.46)	<0.001
**Obesity**	Thoracolumbar	1.71 (1.27 - 2.30)	<0.001
**Coronary artery disease**	Cervical	1.90 (1.25 - 2.90)	0.003
**Coronary artery disease**	Thoracolumbar	1.53 (0.94 - 2.48)	0.085
**Hypothyroidism**	Cervical	1.13 (0.70 - 1.83)	0.619
**Hypothyroidism**	Thoracolumbar	1.16 (0.73 - 1.83)	0.529
**Pituitary dysfunction**	Cervical	6.78 (2.31 - 19.89)	<0.001
**Pituitary dysfunction**	Thoracolumbar	2.89 (0.73 - 11.47)	0.130
**Diabetes mellitus**	Cervical	1.33 (0.98 - 1.79)	0.064
**Diabetes mellitus**	Thoracolumbar	1.16 (0.85 - 1.60)	0.347
**Adrenal insufficiency**	Cervical	7.88 (3.14 - 19.78)	<0.001
**Adrenal insufficiency**	Thoracolumbar	4.43 (1.53 - 12.81)	0.006
**Erectile dysfunction**	Cervical	1.84 (1.13 - 3.01)	0.015
**Erectile dysfunction**	Thoracolumbar	3.88 (2.59 - 5.80)	<0.001
**Depression**	Cervical	2.14 (1.64 - 2.79)	<0.001
**Depression**	Thoracolumbar	2.18 (1.68 - 2.84)	<0.001
**Bipolar disorder**	Cervical	0.23 (0.03 - 1.68)	0.147
**Bipolar disorder**	Thoracolumbar	0.48 (0.13 - 1.81)	0.281
**Schizophrenia psychosis**	Cervical	1.96 (1.05 - 3.65)	0.033
**Schizophrenia psychosis**	Thoracolumbar	1.91 (1.03 - 3.53)	0.041
**Anxiety disorder**	Cervical	2.24 (1.76 - 2.85)	<0.001
**Anxiety disorder**	Thoracolumbar	2.38 (1.86 - 3.04)	<0.001
**Sleep disorder**	Cervical	2.08 (1.58 - 2.74)	<0.001
**Sleep disorder**	Thoracolumbar	1.85 (1.39 - 2.46)	<0.001
**Suicide ideation intent attempt**	Cervical	5.44 (2.94 - 10.06)	<0.001
**Suicide ideation intent attempt**	Thoracolumbar	3.24 (1.59 - 6.58)	0.001
**Substance misuse**	Cervical	1.76 (1.07 - 2.89)	0.025
**Substance misuse**	Thoracolumbar	2.98 (2.00 - 4.44)	<0.001
**Opioid misuse**	Cervical	9.81 (5.54 - 17.38)	<0.001
**Opioid misuse**	Thoracolumbar	11.34 (6.55 - 19.65)	<0.001
**Alcohol misuse**	Cervical	0.93 (0.44 - 1.94)	0.838
**Alcohol misuse**	Thoracolumbar	0.90 (0.44 - 1.87)	0.787
**Ischemic stroke TIA**	Cervical	1.54 (0.84 - 2.83)	0.160
**Ischemic stroke TIA**	Thoracolumbar	1.30 (0.67 - 2.53)	0.444
**Dementia**	Cervical	2.54 (1.28 - 5.08)	0.008
**Dementia**	Thoracolumbar	1.25 (0.56 - 2.77)	0.583
**Seizure disorder**	Cervical	3.21 (1.81 - 5.70)	<0.001
**Seizure disorder**	Thoracolumbar	1.10 (0.47 - 2.61)	0.820

© 2025 Mashlah A et al. *JAMA Network Open*.

Adjusted for age, sex and race. Reference = Uninjured group

eTable 11. Hazard Ratio of Comorbidities Developing After TSCI Stratified by Spine Injury Location in the University of California Cohort

© 2025 Mashlah A et al. *JAMA Network Open*.

eTable 12. Association Between TSCI and Mortality in MGB Cohort.

	None (N = 3114)	SCI (N = 1038)	p-value
**Mortality**	295 (9.5%)	142 (13.7%)	<0.001
**Time to death in years (Median [IQR])**	5.81 (2.18-10.76)	5.7 (2.63-9.84)	0.415
**Age at death in years (Median [IQR])**	76.39 (60.65-86.52)	65.27 (51.65-81.63)	<0.001
Reference= unexposed group<br>Chi-squared performed for mortality, t-test performed for time and age at death			

© 2025 Mashlah A et al. *JAMA Network Open*.

eTable 13. Logistic Regression Analysis of Associations Between Post–Spinal Cord Injury Comorbidities and Mortality in MGB Cohort—Adjusted for Age, Sex and Race.

Comorbidity	Odds Ratio (95%CI)	P-value
**Cardiovascular**		
Hypertension	2.01 (1.16–3.47)	0.012
Hyperlipidemia	0.65 (0.33–1.19)	0.175
Obesity	1.19 (0.52–2.47)	0.655
CAD	1.89 (0.92–3.71)	0.072
**Endocrine**		
Hypothyroidism	2.09 (0.85–4.77)	0.090
Pituitary dysfunction	6.52 (1.14–33.21)	0.025
DM	1.39 (0.51–3.33)	0.488
Adrenal insufficiency	4.98 (1.04–20.20)	0.029
Erectile dysfunction	NA	NA
**Psychiatric**		
Depression	2.90 (1.61–5.15)	<0.001
Bipolar disorder	2.65 (0.39–10.61)	0.223
Schizophrenia/psychosis	5.48 (2.13–13.42)	<0.001
Anxiety disorder	1.95 (1.07–3.48)	0.025
Sleep disorder	1.05 (0.51–2.03)	0.883
Suicide ideation/intent/attempt	2.91 (0.42–12.16)	0.190
Substance misuse	4.02 (1.53–9.84)	0.003
Opioid misuse	2.64 (0.98–6.42)	0.040
Alcohol misuse	4.17 (1.74–9.46)	<0.001
**Neurologic**		
Ischemic stroke/TIA	1.82 (0.81–3.86)	0.131
Dementia	4.81 (1.97–11.59)	<0.001
Seizure disorder	6.38 (2.72–14.47)	<0.001

© 2025 Mashlah A et al. *JAMA Network Open*.

eTable 14. Percentages and Hazard Ratios for Multisystem Comorbidities Developing After TSCI and Time to Development of Comorbidities After Index Date or TSCI Diagnosis, in TSCI and Control Groups in the MBG Cohort

Diagnosis	Uninjured Group N=3114	TSCI Group N=1038	HR (95% CI)	Median time to diagnosis - Uninjured (Years, IQR)	Median time to diagnosis - TSCI (Years, IQR)
**Cardiovascular**					
Hypertension	372 (12%)	169 (16%)	1.58 (1.32-1.90)*	6.82 (3.09-12.08)	7.04 (3.57-11.36)
Hyperlipidemia	121 (3.9%)	61 (5.9%)	1.52 (1.26-1.83)*	5.70 (3.02-10.82)	6.56 (3.53-12.41)
Obesity	362 (12%)	163 (16%)	1.95 (1.49-2.54)*	7.31 (3.76-14.40)	9.00 (4.50-12.79)
Coronary artery disease	143 (4.6%)	89 (8.6%)	1.80 (1.32-2.46)*	7.30 (3.65-13.18)	8.50 (5.52-13.87)
**Endocrine**					
Hypothyroidism	99 (3.2%)	48 (4.6%)	1.48 (1.05-2.10)	7.33 (4.02-10.65)	6.20 (2.94-10.14)
Pituitary dysfunction	13 (0.4%)	8 (0.8%)	1.85 (0.76-4.48)	12.79 (5.98-15.16)	7.60 (6.66-9.66)
Diabetes Mellitus	110 (3.5%)	52 (5.0%)	1.49 (1.07-2.08)	6.77 (2.84-12.23)	8.34 (4.74-13.41)
Adrenal insufficiency	14 (0.4%)	11 (1.1%)	2.50 (1.13-5.53)	6.69 (3.63-12.35)	6.50 (4.18-8.79)
Erectile dysfunction	45 (1.4%)	25 (2.4%)	1.84 (1.12-3.01)	7.14 (3.40-14.18)	4.56 (3.13-12.38)
**Psychiatric**					
Depression	153 (4.9%)	124 (12%)	2.69 (2.12-3.42)*	6.09 (3.38-11.20)	5.53 (3.40-11.46)
Bipolar disorder	16 (0.5%)	19 (1.8%)	3.59 (1.82-7.10)*	9.27 (3.73-17.03)	8.32 (2.42-12.58)
Schizophrenia/psychosis	23 (0.7%)	31 (3.0%)	4.11 (2.39-7.07)*	10.72 (3.92-12.88)	7.29 (2.81-12.04)
Anxiety disorder	235 (7.5%)	161 (16%)	2.26 (1.85-2.77)*	6.88 (3.40-12.77)	6.96 (3.39-11.84)
Sleep disorder	170 (5.5%)	102 (9.8%)	1.97 (1.53-2.52)*	7.43 (3.53-13.43)	9.71 (5.43-14.51)
Suicide ideation/intent/attempt	22 (0.7%)	14 (1.3%)	2.16 (1.08-4.30)	9.00 (5.52-12.39)	14.93 (9.37-18.69)
Substance misuse	41 (1.3%)	42 (4.0%)	3.35 (2.16-5.17)*	6.58 (3.97-13.02)	8.05 (3.15-13.20)
Opioid misuse	17 (0.5%)	37 (3.6%)	6.65 (3.74-11.85)*	6.58 (2.68-12.61)	9.17 (3.77-13.72)
Alcohol misuse	53 (1.7%)	40 (3.9%)	2.39 (1.58-3.63)*	7.46 (4.58-11.81)	9.40 (4.26-14.50)
**Neurologic**					
Ischemic stroke/TIA	64 (2.1%)	46 (4.4%)	2.54 (1.73-3.74)*	7.75 (3.40-14.13)	7.41 (5.17-11.14)
Dementia	37 (1.2%)	34 (3.3%)	3.05 (1.83-5.08)*	10.77 (3.89-16.97)	9.57 (5.96-15.19)
Seizure disorder	36 (1.2%)	29 (2.8%)	2.87 (1.79-4.59)*	5.36 (2.81-13.81)	8.24 (3.34-13.40)

© 2025 Mashlah A et al. *JAMA Network Open*.

eFigure 1. Study Flow Chart for the Mass General Brigham (MGB) and the University of California (UC) Cohorts

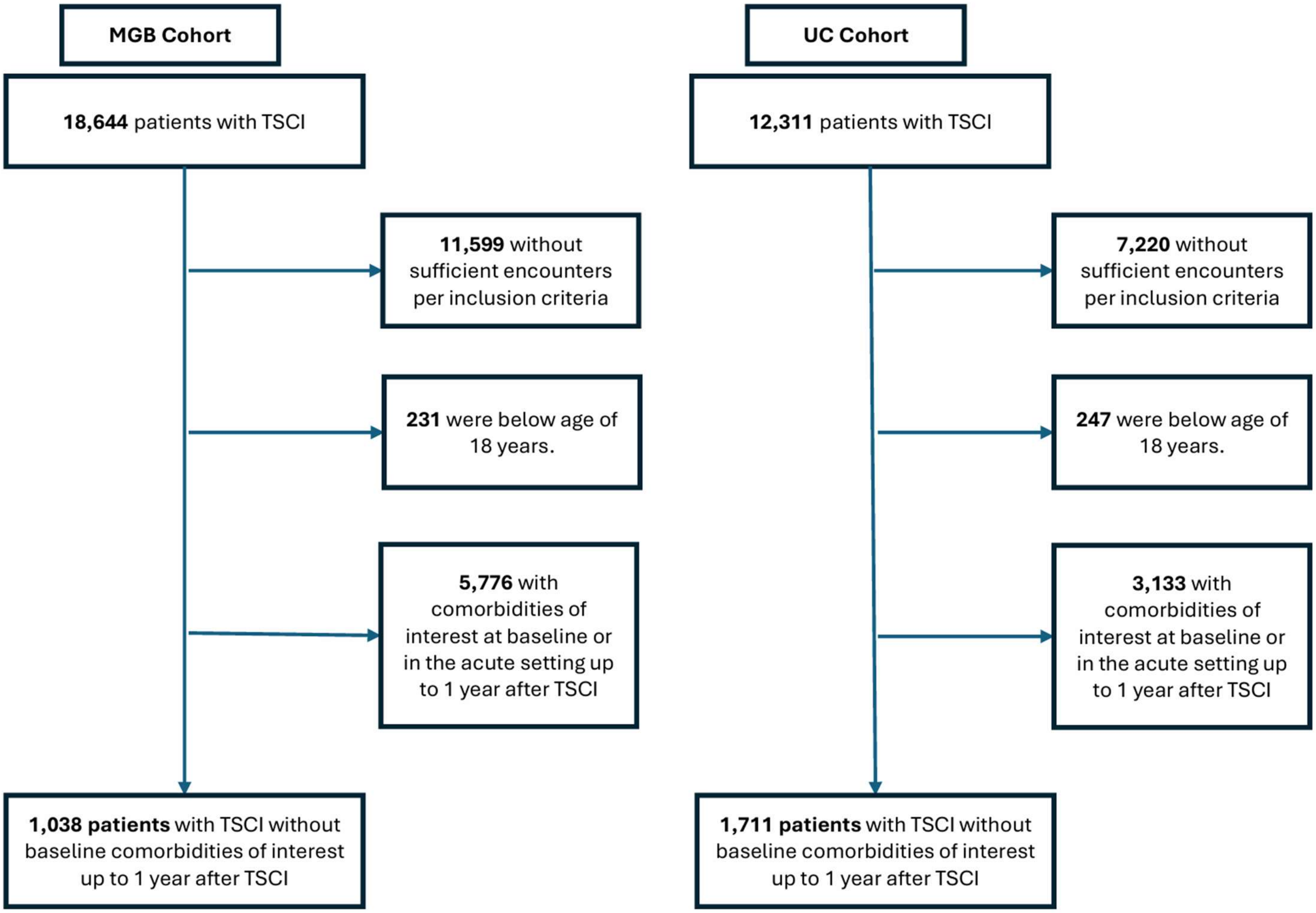

© 2025 Mashlah A et al. *JAMA Network Open*.

e**Figure 2.** Kaplan-Meier Curves of Risk of Multisystemic Comorbidities (Excluded From Figure 2) After TSCI in the Mass General Brigham Cohort

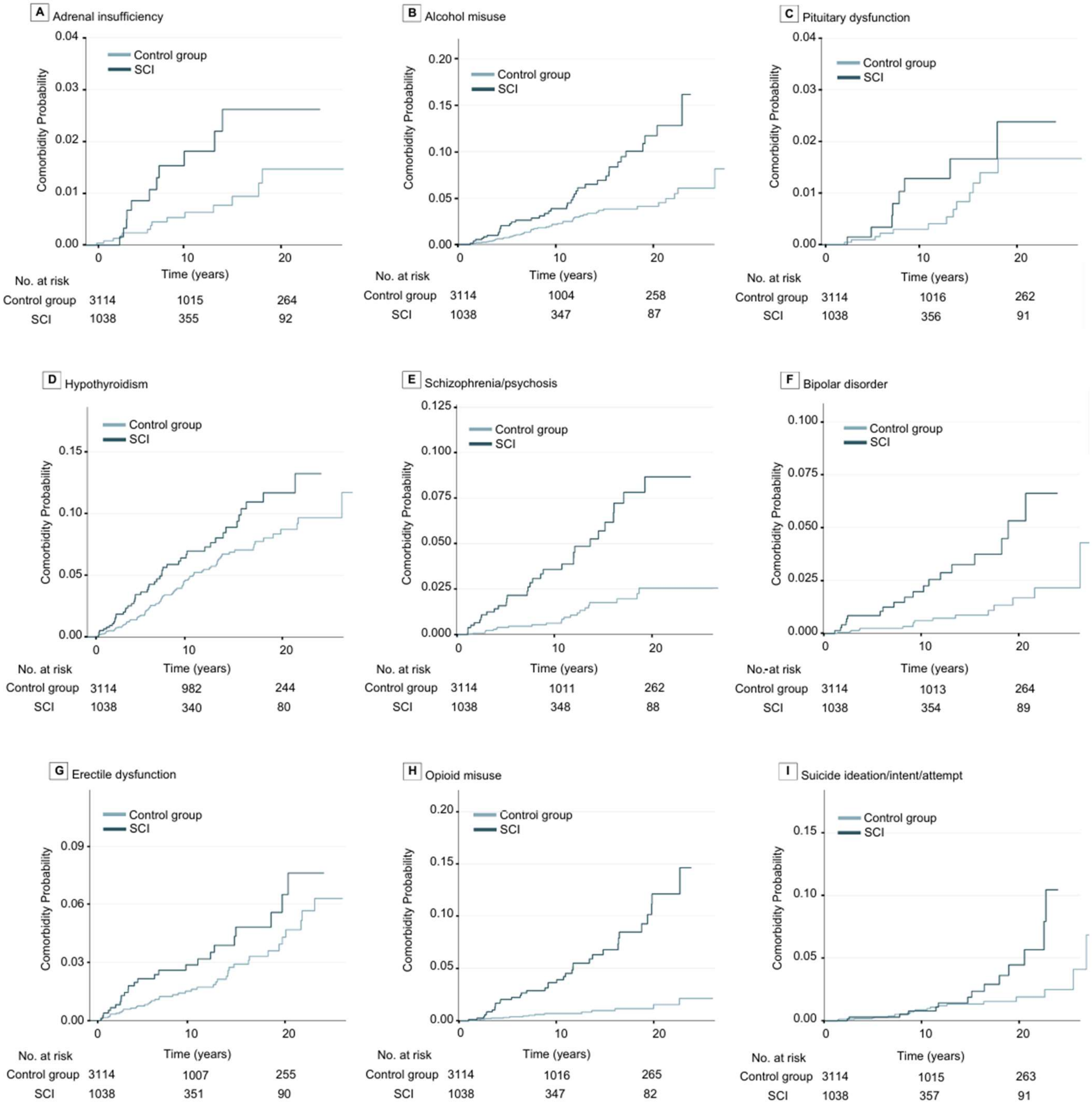

© 2025 Mashlah A et al. *JAMA Network Open*.

eFigure 2–Continued. Kaplan-Meier Curves of Risk of Multisystemic Comorbidities (Excluded From Figure 2) After TSCI in the Mass General Brigham Cohort

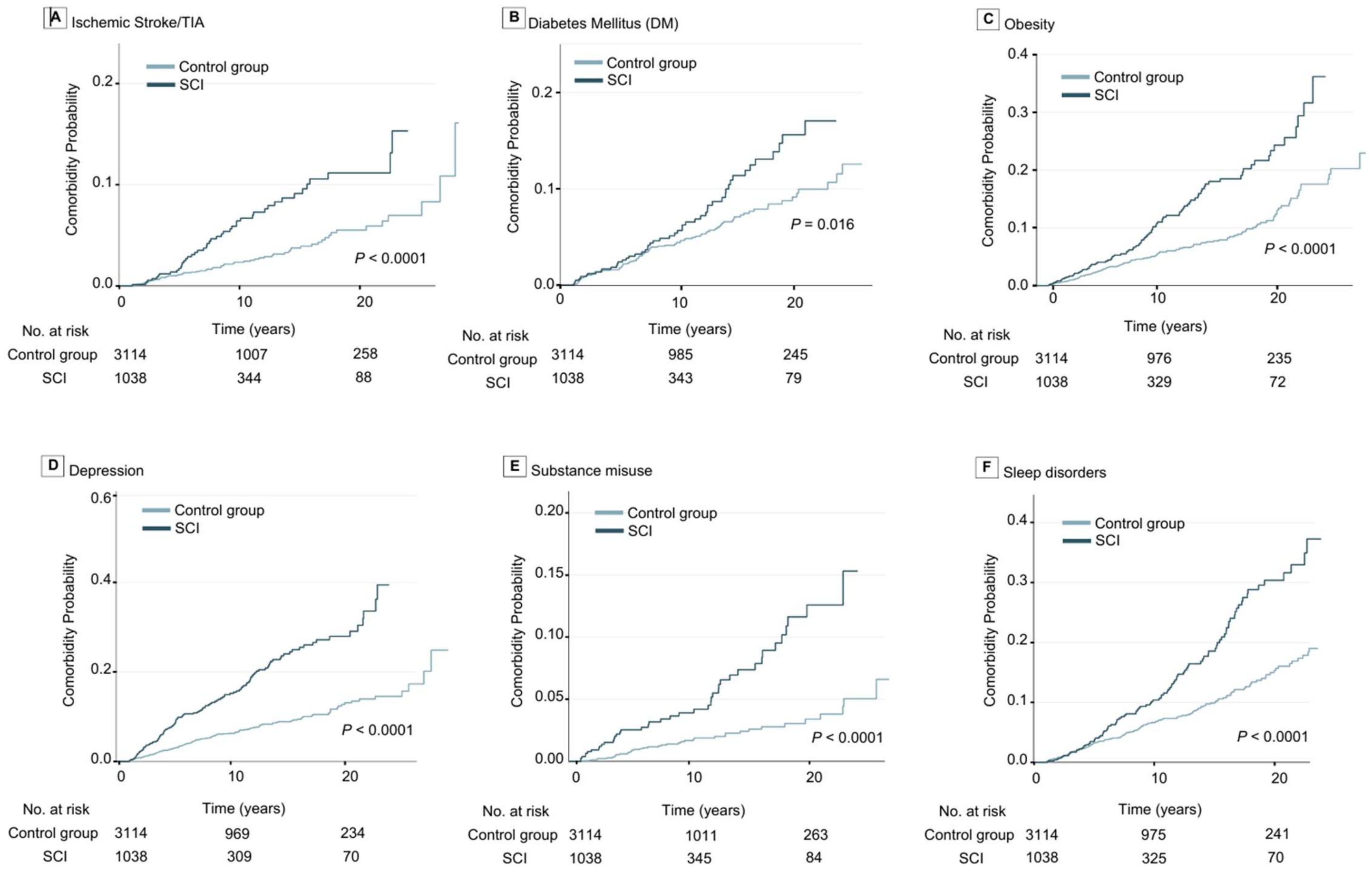

© 2025 Mashlah A et al. *JAMA Network Open*.

eFigure 3. Kaplan-Meier Curves of Risk of the Multisystemic Comorbidities After TSCI in the University of California (UC) Cohort

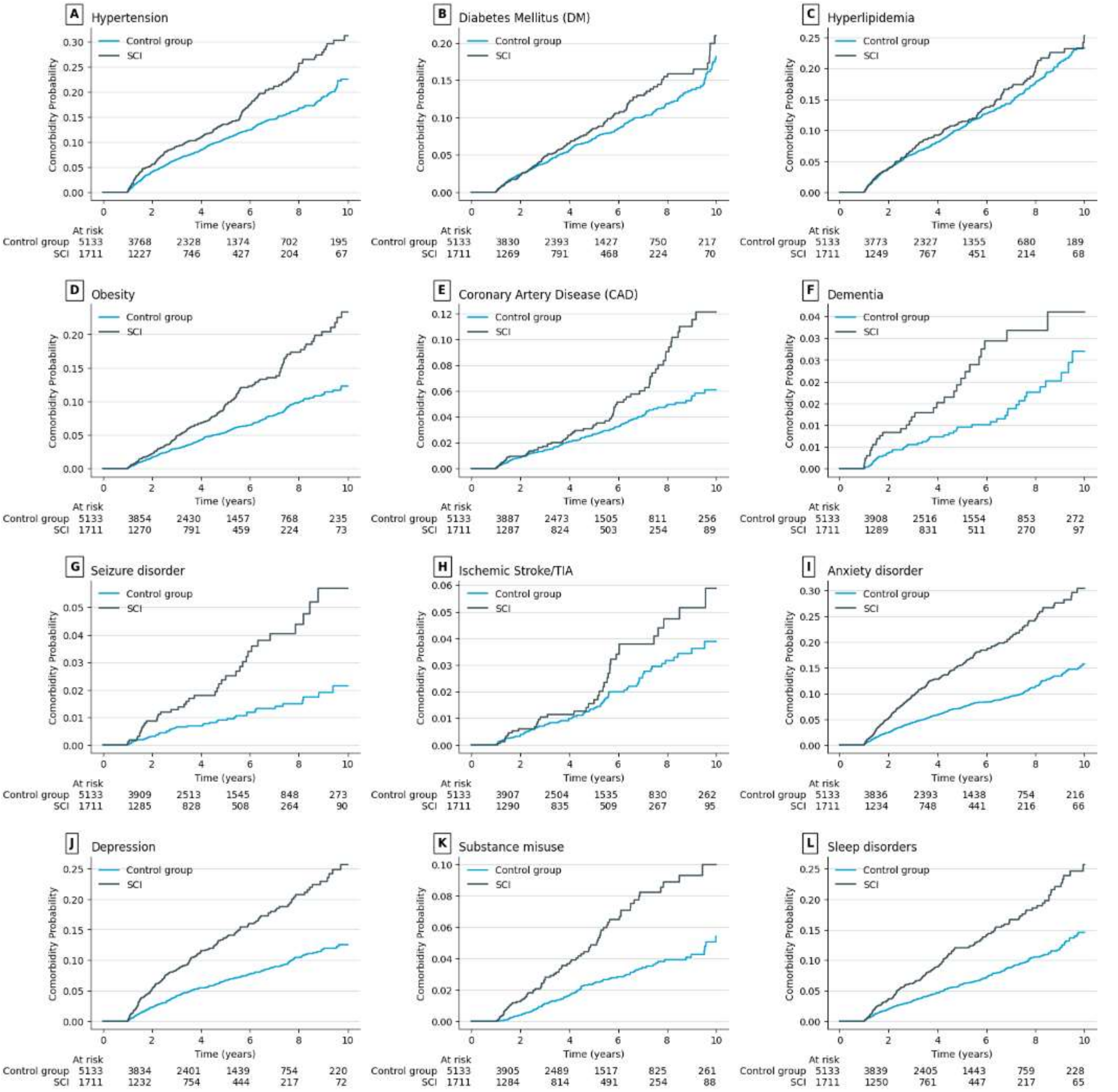

© 2025 Mashlah A et al. *JAMA Network Open*.
