# Supplementary material for: Traumatic Spinal Cord Injury and Subsequent Risk of Developing Chronic Cardiovascular, Neurologic, Psychiatric, and Endocrine Disorders
Source: JAMA Netw Open. 2025 Nov 4;8(11):e2541157. doi: 10.1001/jamanetworkopen.2025.41157 (PMC12587198; doi:10.1001/jamanetworkopen.2025.41157)
Supplement: Supplement 2. — Data Sharing Statement [file jamanetwopen-e2541157-s002.pdf]

Data Sharing Statement

Mashlah. Traumatic Spinal Cord Injury and Subsequent Risk of Developing Chronic Disorders. *JAMA Netw Open*. Published November 04, 2025. doi:10.1001/jamanetworkopen.2025.41157

Data
Data available: No
